# Photocatalytic Pt(IV)‐Coordinated Carbon Dots for Precision Tumor Therapy

**DOI:** 10.1002/advs.202205106

**Published:** 2022-10-28

**Authors:** Dongbo Guo, Josh Haipeng Lei, Dade Rong, Tesen Zhang, Bohan Zhang, Zikang Tang, Han‐Ming Shen, Chu‐Xia Deng, Songnan Qu

**Affiliations:** ^1^ Joint Key Laboratory of the Ministry of Education Institute of Applied Physics and Materials Engineering University of Macau Taipa Macau SAR China; ^2^ School of Biomedical Engineering State Key Laboratory of Marine Resource Utilization in South China Sea Hainan University 570228 Haikou China; ^3^ Faculty of Health Sciences University of Macau Taipa Macau SAR China; ^4^ MOE Frontier Science Centre for Precision Oncology Cancer Center Faculty of Health Sciences University of Macau Taipa Macau SAR China; ^5^ Department of Physics and Chemistry University of Macau Taipa Macau SAR China

**Keywords:** carbon dots, immunogenetic cell death, photocatalyst, Pt(IV) enriched prodrug, tumor therapy

## Abstract

Rapid, efficient, and precise cancer therapy is highly desired. Here, this work reports solvothermally synthesized photoactivatable Pt(IV)‐coordinated carbon dots (Pt‐CDs) and their bovine serum albumin (BSA) complex (Pt‐CDs@BSA) as a novel orange light‐triggered anti‐tumor therapeutic agent. The homogeneously distributed Pt(IV) in the Pt‐CDs (Pt: 17.2 wt%) and their carbon cores with significant visible absorption exhibit excellent photocatalytic properties, which not only efficiently releases cytotoxic Pt(II) species but also promotes hydroxy radical generation from water under orange light. When triggered with a 589 nm laser, Pt‐CDs@BSA possesses the ultrastrong cancer cell killing capacities of intracellular Pt(II) species release, hydroxyl radical generation, and acidification, which induce powerful immunogenic cell death. Activation of Pt‐CDs@BSA by a single treatment with a 589 nm laser effectively eliminated the primary tumor and inhibited distant tumor growth and lung metastasis. This study thus presents a new concept for building photoactivatable Pt(IV)‐enriched nanodrug‐based CDs for precision cancer therapy.

## Graphical abstract

A solvothermally synthesized photoactivatable Pt(IV)‐coordinated carbon dots (Pt‐CDs) and their bovine serum albumin (BSA) complex (Pt‐CDs@BSA) as a novel orange light‐triggered anti‐tumour therapeutic agent with significant visible absorption exhibit excellent photocatalytic properties, which possess ultrastrong cancer cell killing capacities of intracellular cytotoxic Pt(II) species release, hydroxyl radical generation, and acidification, which induce powerful immunogenic cell death (ICD).

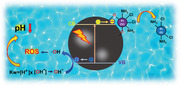

## 1 Introduction

Precision cancer therapy should have all the advantages of efficient tumor targeting and killing, a short treatment time with low chemoresistance, low trauma, and side effects, and of anticancer immune response activation throughout the body. Platinum (Pt)‐based antineoplastic drugs, such as cisplatin, oxaliplatin, and carboplatin, are widely used as first‐line anticancer chemotherapeutic agents for almost all types of cancers.^[^ ^1^ ^]^ However, their therapeutic outcomes are limited by their toxic side effects, as well as by the development of chemoresistance.^[^ ^2^ ^]^ Less toxic Pt(IV) complexes are generally considered to be prodrugs that can undergo reduction by certain agents, such as glutathione (GSH), to produce the corresponding highly cytotoxic Pt(II) species. Although the concentrations of GSH in mammalian cancer tissues are usually much higher than those in normal tissues, there is still a potential risk of the GSH‐triggered Pt(IV) prodrug causing damage to normal tissue.^[^ ^3^ ^]^

The development of photoreducible Pt(IV) prodrugs for controllable Pt(II) production is particularly appealing because it can precisely control Pt(II) species release by external light irradiation at the tumor site in a highly spatial and temporal manner.^[^ ^4^ ^]^ Although some low molecular weight Pt(IV)‐enriched prodrugs that are photoreducible under visible light have been developed,^[^ ^5^ ^]^ the disadvantages of fast metabolism, low accumulation in tumors, and short therapeutic windows have not yet been well addressed. To prolong the blood circulation and tumor accumulation of prodrugs, Pt(IV)‐enriched nanoparticles, including carbon nanotubes, carbon nanoparticles, gold nanoparticles, quantum dots, upconversion nanoparticles, and polymeric micelles, have been designed to deliver Pt(IV) complexes.^[^ ^6^ ^]^ However, the reported Pt(IV)‐enriched nanoparticles have always been reducible under UV light, which has low tissue penetration and high phototoxicity to normal cells and has thus greatly impeded their clinical application.^[^ ^6^, ^7^ ^]^ Moreover, reported photoreducible Pt(IV)‐enriched nanosystems or photodynamic therapy (PDT) with stimulation of antitumor immunity are always limited by single therapy mode with releasing either Pt species or reactive oxygen species (ROS) under photoexcitation,^[^ ^8^ ^]^ which might require several rounds of treatment to induce robust antitumor immunity. Hence, an efficient photoreducible Pt(IV) nanosystem with simultaneous ROS generation under longer wavelength excitation to induce robust antitumor immunity and long therapeutic windows is highly demanded.

Carbon dots (CDs) are 0D quasi‐spherical particles composed of sp^2^/sp^3^ hybridized carbon atoms with unique photophysical and chemical properties, such as light absorption over a wide range, bright photoluminescence (PL), high aqueous solubility, low toxicity, chemical inertness, and excellent photocatalysis potential.^[^ ^9^ ^]^ CDs contain abundant functional groups, such as −COOH and −OH, which can be sites to realize photoactivatable Pt(IV)‐coordinated CDs under visible light.^[^ ^10^ ^]^ To the best of our knowledge, there are no reported photoactivatable Pt(IV)‐coordinated CDs for tumor therapy.

In this work, for the first time, we developed a new type of Pt(IV)‐coordinated CDs (Pt‐CDs) from o‐phenylenediamine and oxoplatin by solvothermal treatment in *N*,*N*’‐dimethylformamide (DMF). The prepared Pt‐CDs have a Pt(IV) content of approximately 17.2 wt% with a broad absorption band in the visible region and low cytotoxicity in the dark. The carbon cores of Pt‐CDs act as not only visible light absorbers but also excellent photocatalysts, in which the photogenerated electrons reduce Pt(IV) to Pt(II) and the photogenerated holes oxidize hydroxyl ions to generate hydroxy radicals with acidification in aqueous solution. After the incorporation of bovine serum albumin (BSA), the BSA composite Pt‐CDs (Pt‐CDs@BSA) exhibited larger particle sizes of 50–120 nm with much higher cellular uptake and tumor accumulation than pure Pt‐CDs. In vitro cell culture studies demonstrated that the endocytosed Pt‐CDs@BSA in cancer cells can quickly release toxic Pt(II) species, generate hydroxyl radicals, and cause intracellular acidification upon radiation with a 589 nm laser, leading to effective immunogenic cell death (ICD). Moreover, we used a 4T1 cell‐derived orthotopic breast tumor mouse model and found that Pt‐CDs@BSA accumulated in tumors after only one round of intravenous treatment and not only eliminated the primary tumor but also inhibited the growth of distant tumors and cancer metastasis in the lung, indicating the superior antitumor and antimetastatic efficacy of Pt‐CDs@BSA. Thus, our study presents a novel CD‐based Pt(IV)‐enriched therapeutic agent for precision tumor therapy.

## 2 Results and Discussion

### 2.1 Synthesis and Characterization of Pt‐CDs

**Figure 1:**
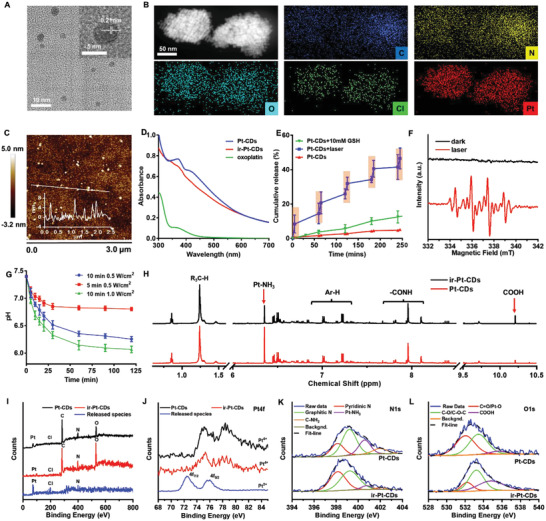
Morphological, structural, and photochemical characterization of Pt(IV)‐coordinated carbon dots (Pt‐CDs). A) Transmission electron microscopy (TEM) and high‐resolution TEM (HRTEM) (inset) images of Pt‐CDs. B) Scanning transmission electron microscopy (STEM) image of Pt‐CDs and the corresponding energy‐dispersive X‐ray spectroscopy (EDX) element maps of C, N, O, Cl, and Pt. C) Atomic force microscopy (AFM) image of Pt‐CDs. Inset: height profile along the line. D) Absorption spectra of oxoplatin, Pt‐CDs, and ir‐Pt‐CDs at 0.25 mg mL^−1^ in H_2_O. E) Pt release profiles from 0.25 mg mL^−1^ Pt‐CDs aqueous solutions with 10 × 10^−3^ m glutathione (GSH) with or without 589 nm laser irradiation at 0.5 W cm^−2^. The time for each laser irradiation was 5 min. F) Electron spin resonance (ESR) spectra of DMPO‐OH adducts formed in Pt‐CDs with or without laser irradiation. [DMPO] = 0.75 × 10^−3^ m, [Pt‐CDs] = 0.25 mg mL^−1^; *T* = 298 K. G) pH variations of 0.25 mg mL^−1^ Pt‐CDs aqueous solutions under 5 min or 10 min of 589 nm laser irradiation at 0.5 or 1.0 W cm^−2^. H) ^1^H NMR spectra of Pt‐CDs and ir‐Pt‐CDs in DMSO‐*d_6_*. I) X‐ray photoelectron spectroscopy (XPS) survey spectra and J) high‐resolution Pt4f spectra of Pt‐CDs, ir‐Pt‐CDs, and the released species. High‐resolution (K) N1s and (L) O1s spectra of Pt‐CDs and ir‐Pt‐CDs.

Oxoplatin was prepared according to our previous report.^[^ ^6^, ^11^ ^]^ Pt(IV)‐coordinated CDs (Pt‐CDs) were synthesized from o‐phenylenediamine and oxoplatin in DMF under solvothermal conditions at 200 °C. The morphology and structure of the Pt‐CDs were investigated by transmission electron microscopy (TEM) and atomic force microscopy (AFM). As shown in **Figure** **1**A,C and Figure S1 (Supporting Information), the Pt‐CDs showed a homogeneous sphere‐like morphology with sizes from 3 to 6 nm. The high‐resolution TEM (HRTEM) image of the Pt‐CDs revealed lattice fringes of 0.21 nm, corresponding to the (100) crystal plane of graphitic carbon (inset of Figure 1A). X‐ray photoelectron spectroscopy (XPS) analyses revealed that the Pt‐CDs consisted of C, N, O, Cl and Pt elements with weight contents of 53.0, 8.9, 14.9, 6.0, and 17.2 wt%, respectively, indicating Pt enrichment (Table S1, Supporting Information). In the scanning transmission electron microscopy (STEM) and energy dispersive X‐ray spectrometry (EDX) maps, the C, N, Pt, Cl, and O elements were homogeneously distributed in the sample, indicating that the Pt atoms in the Pt‐CDs were homogeneously coordinated (Figure 1B).

### 2.2 Photoactivation Properties of Pt‐CDs

Pt‐CDs show strong broad absorption bands from the UV to visible regions with tails up to the near‐infrared region, while oxoplatin exhibits absorption bands in only the UV region (Figure 1D). The photoactivation of Pt‐CDs was investigated using a 589 nm laser. As shown in Figure 1E, Pt‐CDs exhibited a significant orange light‐triggered Pt release profile. Approximately 46.5% of the Pt was released from the Pt‐CDs after five cycles of 589 nm laser irradiation at 0.5 W cm^−2^ for 5 min each time. It can be seen that the cumulative laser‐induced Pt release after 25 min is much higher than that from the Pt‐CDs aqueous solution treated with 10 × 10^−3^ m GSH for 4 h (Figure S2, Supporting Information), indicating that orange light‐triggered Pt release from Pt‐CDs was much more efficient than GSH‐induced Pt release. The sample after 30 min of 589 nm laser irradiation (0.5 W cm^−2^) followed by dialysis purification was named ir‐Pt‐CDs. The absorption band of ir‐Pt‐CDs in the UV region was slightly decreased with blueshifted fluorescence (FL) (Figure 1D; Figures S3 and S4, Supporting Information).

The orange light‐triggered Pt release indicated that a photocatalytic reaction had occurred in the Pt‐CDs, in which the photogenerated electron and hole pairs in the carbon cores separated and induced reduction and oxidation reactions, respectively. The photoinduced reduction reaction could be the reason for the Pt species release in which Pt(IV) transforms to Pt(II). Electron spin resonance (ESR) spectra of Pt‐CDs aqueous solutions with DMPO and with and without 589 nm laser irradiation were obtained, as shown in Figure 1F. In the dark, the ESR measurements showed no signal from the Pt‐CDs in aqueous solution. In contrast, typical fourfold signals with ratios of 1:2:2:1, which are from the hydroxyl radicals captured by DMPO, were clearly observed in the Pt‐CDs aqueous solution after 10 min of laser irradiation. This photoinduced hydroxyl radical generation could be from the photoinduced oxidation of OH^−^ to ⋅OH, which can be further reflected by the decreased pH values of the Pt‐CDs aqueous solution after 589 nm laser irradiation (Figure 1G). The increased [H^+^] can be well explained by the decreased [OH^−^] caused by the photoinduced oxidation to form ⋅OH. It should be noted that the decrease in the pH of the Pt‐CDs aqueous solution can be controlled by the laser power and irradiation time. Ten minutes of 589 nm laser irradiation at 0.5 W cm^−2^ on a 0.25 mg mL^−1^ Pt‐CDs aqueous solution can quickly cause a decrease in the pH to below 6.5 (Figure 1G and Figure S5, Supporting Information). This photoinduced Pt release was further demonstrated by ^1^H NMR spectroscopy (Figure 1H). The peaks at 6.4 ppm for Pt‐NH_3_ and peaks at 10.3 ppm for COOH decreased and increased in intensity from Pt‐CDs to ir‐Pt‐CDs, respectively, while the other peaks were nearly unchanged.

The species released from the dialysis bag (1000 MWCO) were collected and freeze‐dried for further XPS study. In comparison with the Pt‐CDs, the weight contents of Pt, Cl, and N in the ir‐Pt‐CDs decreased significantly to 6.0, 3.0, and 9.7 wt%, respectively, while the ratio of C to O decreased from 4.8 in Pt‐CDs to 3.6 in ir‐Pt‐CDs (Figure 1I and Table S1, Supporting Information). The increased O content in the ir‐Pt‐CDs might be due to further surface oxidation by the photogenerated hydroxyl radicals. In contrast, the weight contents of Pt, Cl, and N in the released species were 70.1, 20.7, and 7.5 wt%, respectively, with an atomic ratio of 1:1.62:1.49, while C and O were hardly detected in the released species, indicating that the photoreleased species are Pt‐coordinated small molecules (Table S1, Supporting Information). The high‐resolution Pt4f, N1s, O1s, and C1s spectra of Pt‐CDs, ir‐Pt‐CDs and the released species were further examined, as shown in Figure 1J–L and Figure S6–S8 (Supporting Information). The high‐resolution Pt4f spectra of the Pt‐CDs and ir‐Pt‐CDs showed 4f_5/2_ and 4f_7/2_ peaks at 78.8 and 75.5 eV, respectively, indicating that the contained Pt atoms only existed in the form of Pt(IV) in the two samples. In contrast, the 4f_5/2_ and 4f_7/2_ signals of the released species were observed at only 76.0 and 72.8 eV, respectively, showing the presence of Pt(II) (Figure 1J). Compared with the N1s spectra, the peak at 400.8 eV for (NH_3_)_2_Pt was much lower in the ir‐Pt‐CDs sample than in the Pt‐CDs sample. These results indicated that the orange light absorbed by the Pt‐CDs caused the photoinduced reduction of Pt(IV) in Pt‐CDs to release Pt(II)‐coordinated small molecules (Figure 1K and Table S3, Supporting Information). Compared with the O1s spectrum of the Pt‐CDs, ir‐Pt‐CDs showed a decreased Pt‐O signal at 532 eV and an increased COOH signal at 535.8 eV, indicating that Pt(IV) was coordinated in Pt‐CDs via Pt‐O bonds, which can break during the photoinduced reduction process to form COOH or OH groups and release Pt(II)‐coordinated small molecules (Figure 1L and Table S4, Supporting Information). The increase in the number of COOH groups can also be reflected by the enhanced COOH signals at 291.2 eV in the C1s spectrum of ir‐Pt‐CDs, agreeing well with the ^1^H NMR data. Based on the reference integrations of −CH_3_ at 1.24 ppm, the increased integrations of −COOH (≈10.2 ppm) and decreased integrations of −NH_3_ (≈6.35 ppm) were 19.1 and 58.06, respectively, which was nearly to 1/3 (Table S5, Supporting Information), indicating that one Pt(II) molecule released from Pt‐CDs could lead to two COOH groups of generation and two NH_3_ ligands of disappearance. The results further demonstrated ester bond was the major binding way between the Pt(IV) and carbon dots.

**Figure 2:**
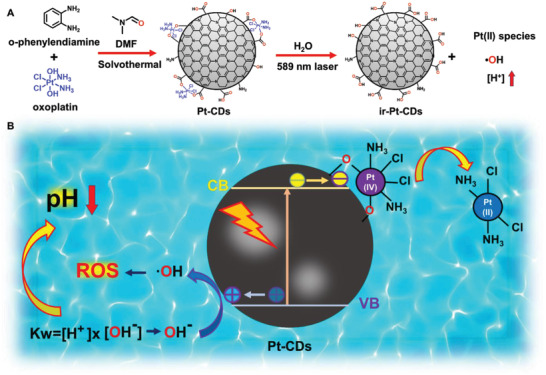
The proposed mechanism of photoreduciable Pt(IV)‐coordinated carbon dots (Pt‐CDs). A) Schematic of the synthesis of Pt‐CDs and their chemical structure changes after 589 nm laser irradiation. B) A proposed mechanism of the photocatalytic processes in the Pt‐CDs in an aqueous environment.

Based on the results above, a proposed photocatalytic mechanism of Pt‐CDs is illustrated in **Figure** **2**. Upon orange light excitation, photoinduced charge separation occurs in the Pt‐CDs. The photogenerated electrons in the conduction band (CB) of the carbon cores are captured by the coordinated Pt(IV) and undergo a reduction reaction to release Pt(II) species. The photogenerated holes in the valence band (VB) of the carbon cores interact with water and oxidize with OH^−^ to generate hydroxyl radicals. The photoinduced consumption of OH^−^ breaks the balance between [OH^−^] and [H^+^], leading to increased [H^+^] and decreased pH values. All of these results indicated that Pt‐CDs exhibit excellent photocatalysis under orange light irradiation to efficiently release Pt(II) species and generate hydroxyl radicals accompanied by pH value decline in aqueous environments.

### 2.3 Composition of Pt‐CDs and BSA

**Figure 3:**
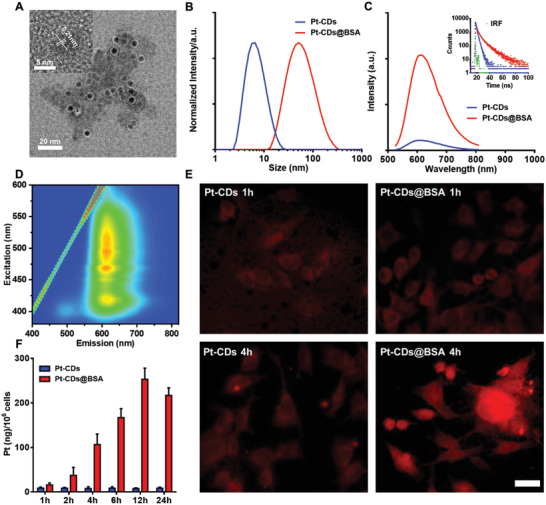
The morphology, size, optical properties, and cellular uptake of Pt‐CDs@BSA. A) Transmission electron microscopy (TEM) and high‐resolution TEM (HRTEM) (inset) images of Pt‐CDs@BSA. B) Dynamic light scattering (DLS) profiles of Pt‐CDs and Pt‐CDs@BSA in aqueous solutions. C) Photoluminescence (PL) spectra of Pt‐CDs and Pt‐CDs@BSA in dilute aqueous solutions at 520 nm excitation. Inset: fluorescence (FL) decay curves of Pt‐CDs and Pt‐CDs@BSA in aqueous solutions monitored at 600 nm under 512 nm excitation. D) Excitation‐emission map of Pt‐CDs@BSA in aqueous solution. E) FL images of 4T1 cells treated with Pt‐CDs and Pt‐CDs@BSA at a Pt content of 10 × 10^−6^ m. Scale bar = 20 µm. F) Cellular uptake of Pt‐CDs and Pt‐CDs@BSA by 4T1 cells after 1–24 h of incubation. The amount of Pt per cell was determined by inductively coupled plasma‒mass spectrometry (ICP‒MS). Data presented as mean ± Standard Deviation (SD) (*n* = 5).

Pt‐CDs exhibit ultrasmall particle sizes (3–6 nm) and weak FL in aqueous solution. It has been demonstrated in our previous work that the surface of carbon dots complexed with BSA can effectively prevent water molecules to reduce the energy dissipation, causing the efficient emission in aqueous solutions.^[^ ^12^ ^]^ To enlarge the particle size and enhance the FL in aqueous media, BSA was incorporated into the Pt‐CDs. Pt‐CDs and BSA complexes (Pt‐CDs@BSA) were fabricated by heating 1.5 mg of Pt‐CDs and 30 mg of BSA in 6 mL of water at 50 °C for 1 h. Excitingly, the Pt‐CDs@BSA particle size was significantly enlarged to dozens of nanometers (**Figure** **3**B). The TEM image revealed that the Pt‐CDs@BSA were composed of several Pt‐CDs entangled by BSA (Figure 3A). Circular dichroism spectral analysis exhibited little change in the conformation of BSA (Figure S9, Supporting Information). Considering the moderate thermal treatment, it can be inferred that Pt‐CDs are entangled by BSA to form Pt‐CDs@BSA clusters. Pt‐CDs@BSA inherit the broad absorption band in the visible region with slightly enhanced absorption bands in the UV region (Figure S10, Supporting Information). In contrast to the weak FL from Pt‐CDs, Pt‐CDs@BSA exhibit enhanced red FL (9.5‐fold) in aqueous solution with photoluminescence quantum yields (PLQYs) of 10.8% under 520 nm excitation (Figure 3C and Figure S11, Supporting Information). The average FL lifetime of Pt‐CDs@BSA in aqueous solution monitored at 600 nm under 512 nm excitation was 8.5 ns, which is much longer than that of Pt‐CDs in aqueous solution (2.2 ns), indicating that complexation with BSA can effectively prevent the FL quenching of Pt‐CDs by water molecules. The excitation‐emission map indicated that Pt‐CDs@BSA exhibited excitation‐independent red FL with an emission center at 610 nm (Figure 3D).

### 2.4 Cellular Uptake and In Vitro Cytotoxicity of Pt‐CDs and Pt‐CDs@BSA

The biological activities of Pt‐CDs and Pt‐CDs@BSA were subsequently investigated. We incubated 4T1 cells with Pt‐CDs and Pt‐CDs@BSA and examined their red FL at 1, 2, 4, and 6 h (Figure 3E and Figure S12, Supporting Information). Our results showed that the red FL intensity significantly increased in the Pt‐CDs@BSA group compared with the Pt‐CD group at 4 h, suggesting that Pt‐CDs@BSA exhibit higher cellular uptake than Pt‐CDs. Moreover, the subcellular locations of Pt‐CDs@BSA against 4T1 cells were investigated using colocation studies with LysoTracker Green. After 4 h incubation, Pt‐CDs@BSA exhibited good colocalization with lysosomes (Figure S13, Supporting Information), concluding that Pt‐CDs@BSA was internalized by endocytosis. To investigate the cellular uptake capability in detail, we introduced inductively coupled plasma‒mass spectrometry (ICP‒MS) to quantify the cellular uptake of Pt from cisplatin, Pt‐CDs, and Pt‐CDs@BSA after incubation with 4T1 cells at different time points (Figure 3F; Figures S14 and S15, Supporting Information). Our data revealed that the intracellular Pt concentrations dramatically increased with time in the Pt‐CDs@BSA‐treated group. In contrast, there was no significant change in the intracellular Pt concentration in the Pt‐CDs group. The intracellular Pt concentration was 33‐fold and 4‐fold higher in cells incubated with Pt‐CDs@BSA than cells incubated with Pt‐CDs and cisplatin at 12 h, respectively (Table S6, Supporting Information). The reason was that the Pt‐CDs with negative charge (−25 mV) repelled to the cell membrane (Figure S16, Supporting Information), which adhere preferentially to positively charged surfaces. After trapped with BSA, the zeta potential turned into neutral (−5 mV). Hence, the Pt‐CDs@BSA were easily internalized into the cytoplasm within 4 h, as observed by the intracellular FL.^[^ ^13^ ^]^ Low temperature could significantly reduce the internalization of Pt‐CDs@BSA (Figure S15, Supporting Information), substantiating that the cellular uptake of Pt‐CDs@BSA was energy‐dependent endocytosis.

**Figure 4:**
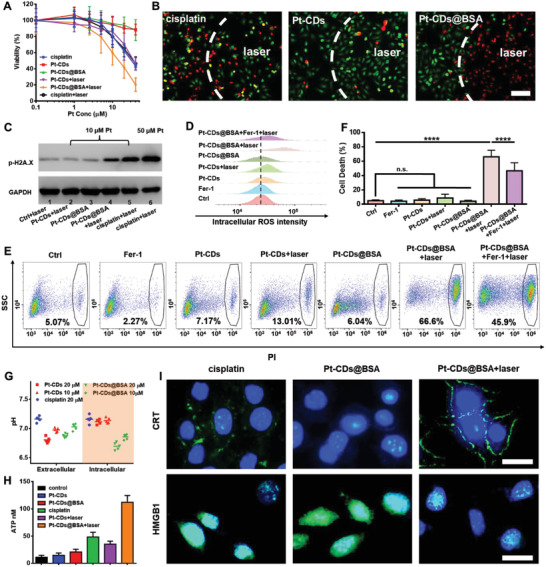
Orange light‐triggered cell‐killing capacities of Pt‐CDs@BSA. A) Cytotoxicity of cisplatin, Pt‐CDs, and Pt‐CDs@BSA to 4T1 cells with or without 589 nm laser irradiation (0.5 W cm^−2^, 10 min) by MTT assay at different Pt concentrations for 48 h. Data represent the mean ± SD (*n* = 5). B) Live/dead viability assays were performed in 4T1 cells with cisplatin, Pt‐CDs, and Pt‐CDs@BSA at a Pt concentration of 10 × 10^−6^ m with and without 589 nm laser irradiation (0.5 W cm^−2^, 10 min). Scale bar: 50 µm. C) p‐Histone H2A.X expression in cisplatin‐, Pt‐CD‐, and Pt‐CD@BSA‐treated 4T1 cells with or without 589 nm laser irradiation (0.5 W cm^−2^, 10 min, 10 × 10^−6^ m Pt) by Western blot analysis. Glyceraldehyde 3‐phosphate dehydrogenase (GAPDH) was used as an internal control. D) Intracellular reactive oxygen species (ROS) intensities of 4T1 cells treated with Fer‐1, Pt‐CDs, Pt‐CDs + laser, Pt‐CDs@BSA, Pt‐CDs@BSA + laser, and Pt‐CDs@BSA + Fer‐1 + laser. [Pt] = 10 × 10^−6^ m, [Fer‐1] = 10 × 10^−6^ m. (E) PI exclusion assay of 4T1 cells treated with Fer‐1, Pt‐CDs@BSA, Pt‐CD@BSA+laser, and Pt‐CDs@BSA+ Fer‐1+laser. Irradiation at 589 nm, 0.5 W cm^−2^, [Pt] = 10 × 10^−6^ m, [Fer‐1] = 10 × 10^−6^ m. F) Statistical analysis of 4T1 cell death in (E) (*n* = 5). *p*‐values are calculated using one‐way ANOVA, n.s.: no statistical significance; ^****^ *p* < 0.001; ^**^ *p* < 0.01. G) pH_i_ and pH_e_ of 4T1 cells loaded with the fluorescent pH indicator BCECF‐AM and incubated with cisplatin, Pt‐CDs, and Pt‐CDs@BSA at a Pt concentration of 10 or 20 × 10^−6^ m with 589 nm light irradiation (0.5 W cm^−2^, 10 min). H) ATP secretion from cisplatin‐ and Pt‐CDs@BSA‐treated 4T1 cells at a Pt concentration of 10 × 10^−6^ m with or without 589 nm laser irradiation. (I) CLSM images of surface‐exposed CRT on 4T1 cells and HMGB1 release from 4T1 cells treated with cisplatin and Pt‐CDs@BSA at a Pt concentration of 10 × 10^−6^ m with and without 589 nm laser irradiation (0.5 W cm^−2^, 10 min). Scale bar = 20 µm. Data presented as mean ± S.D. (*n* = 3).

The cytotoxicity of Pt‐CDs and Pt‐CDs@BSA with or without 589 nm laser irradiation was further evaluated. 4T1 cells were incubated with different doses of Pt‐CDs or Pt‐CDs@BSA for 6 h followed by 589 nm laser irradiation (0.5 W cm^−2^) for 10 min or incubation in the dark. Cell viability was measured by MTT assay after 48 h of incubation. Without irradiation, there was no effect on the cell viability after treatment with Pt‐CDs and Pt‐CDs@BSA, even at a Pt dose of up to 40 × 10^−6^ m (**Figure** **4**A). In contrast, cell viability was significantly decreased after cisplatin treatment at a Pt dose higher than 20 × 10^−6^ m. Moreover, both Pt‐CDs and Pt‐CDs@BSA exhibited enhanced cytotoxicity upon 589 nm laser irradiation, with phototoxic index values as high as 9.1 and 31, respectively (Table S7, Supporting Information). Notably, treatment with Pt‐CDs@BSA demonstrated much higher cytotoxicity than treatment with Pt‐CDs under the same laser irradiation. Furthermore, we employed a live/dead cell viability assay to investigate the orange light‐induced cell death abilities of these agents with a Pt content of 10 × 10^−6^ m (Figure 4B and Figure S17, Supporting Information). No significant cell death occurred in the Pt‐CD, while a clear dividing line between the area of unirradiated cells with green FL and the irradiated cells with red FL was observed in the cells treated with Pt‐CDs@BSA, indicating a significant difference in cell death. It should be noted that the orange light‐induced cell death of Pt‐CDs@BSA was much higher than that of cisplatin (Figure 4B).

Cisplatin‐induced cell death is associated with DNA damage caused by Pt(II).^[^ ^7^ ^]^ Thus, we measured the level of p‐H2A.X, which is phosphorylated and recruited to sites of DNA double‐strand breaks in cisplatin‐, Pt‐CDs‐, and Pt‐CDs@BSA‐treated 4T1 cells with or without 589 nm laser irradiation (Figure 4C).^[^ ^14^ ^]^ The expression of p‐H2A.X remained low in Pt‐CDs‐treated cells with laser irradiation and Pt‐CDs@BSA‐treated cells without laser irradiation, suggesting less DNA damage. In contrast, the expression of p‐H2A.X was clearly upregulated in the Pt‐CD@BSA‐treated cells with laser irradiation. It should be noted that cisplatin with the same Pt concentration (10 × 10^−6^ m) induced much higher expression of p‐H2A.X but displayed lower cell viability than Pt‐CDs@BSA with 589 nm laser irradiation. As the Pt(II) species could not be completely released from Pt‐CDs and Pt‐CDs@BSA after 589 nm laser irradiation with the testing time (Figure 1E and Figure S18, Supporting Information). It can therefore be inferred that the DNA damage caused by the orange light‐induced Pt(II) species released from Pt‐CDs@BSA is not the only factor that causes much more significant cell death than cisplatin.

Singlet oxygen‐related PDT and hydroxyl radical‐related ferroptosis are promising clinical cancer therapies that are dependent on the O_2_ and H_2_O_2_ levels in the tumor microenvironment, respectively. Considering that a significant number of hydroxyl radicals can be generated by Pt‐CDs from water under 589 nm laser irradiation, which does not need O_2_ or H_2_O_2_, the intracellular ROS level in Pt‐CDs@BSA‐treated 4T1 cells after 589 nm laser irradiation was detected by flow cytometry analysis. Pt‐CDs@BSA‐treated 4T1 cells showed higher levels of ROS with 589 nm laser irradiation, which were significantly reduced by the ferroptosis inhibitor ferrostatin‐1 (Fer‐1) (Figure 4D). Consistently, the propidium iodide (PI) exclusion assay showed that Pt‐CDs@BSA could significantly kill 4T1 cells after 589 nm laser irradiation, and Fer‐1 was found to have a significant protective effect (Figure 4E,F). However, both Z‐VAD, a pan‐caspase inhibitor, and *N*‐acetylcysteine (NAC), a well‐known ROS inhibitor, were unable to suppress the cell death induced by Pt‐CDs@BSA (Figure S19, Supporting Information). Considering that Fer‐1 can scavenge hydroxyl radicals to prevent lipid peroxidation of the cellular membrane,^[^ ^15^ ^]^ it is thus believed that the hydroxyl radicals generated from Pt‐CDs@BSA play a key role in the strong orange light‐induced cytotoxicity.

Cancer cells have been reported to have a slightly alkaline intracellular environment with stronger cell proliferation and apoptosis evasion.^[^ ^2^, ^16^ ^]^ It has been demonstrated that an acute reduction in intracellular pH (pH_i_) can be an effective way to induce cell death.^[^ ^17^ ^]^ Considering that the pH values of Pt‐CDs aqueous solution can be significantly decreased by 589 nm laser irradiation accompanied by hydroxyl radical generation, the intracellular pH regulation of Pt‐CDs and Pt‐CDs@BSA‐treated cells with or without laser irradiation was examined with a fluorescent pH probe (2’,7’‐bis‐(2‐carboxyethyl)‐5‐(and‐6)‐carboxyfluorescein acetoxymethyl ester (BCECF‐AM)) (Figure S20, Supporting Information). We found that 4T1 cells treated with Pt‐CDs@BSA at a Pt concentration of 20 × 10^−6^ m after 10 min of 589 nm laser irradiation (0.5 W cm^−2^) resulted in a significant decrease in the pH_i_ and extracellular pH (pH_e_) to values of approximately 6.7 (Figure 4G and Figure S21, Supporting Information). Due to the lower cellular uptake of Pt‐CDs, the pH_i_ of Pt‐CDs‐treated 4T1 cells (Pt concentration of 20 × 10^−6^ m) after the same dose of laser irradiation remained at approximately 7.1, while their pH_e_ decreased to less than 6.7. In contrast, both the pH_i_ and pH_e_ of the cisplatin‐treated 4T1 cells remained at approximately 7.1 after the same dose of laser irradiation. It can therefore be inferred that the much higher orange light‐induced cell death caused by Pt‐CDs@BSA than cisplatin is due to the combined effect of the orange light‐induced Pt(II) species release, hydroxy radical generation, and pH_i_ decrease.

### 2.5 Photoinduced ICD of Pt‐CDs@BSA In Vitro

It has been reported that Pt‐based chemotherapeutic drugs can not only trigger apoptosis but also evoke antitumor immunity responses to some extent by inducing ICD.^[^ ^18^ ^]^ Calreticulin (CRT) exposure, high mobility group box 1 (HMGB1) release, and ATP secretion are always accompanied by the initiation of ICD, and could serve as biomarkers to evaluate ICD efficacy.^[^ ^19^ ^]^ Thus, ATP, CRT and HMGB1 expression in 4T1 cells treated with cisplatin, Pt‐CDs, and Pt‐CDs@BSA at a Pt content of 10 × 10^−6^ m with or without 589 nm laser irradiation was investigated. As shown in Figure 4H, Pt‐CDs@BSA‐treated 4T1 cells irradiated with a 589 nm laser displayed 2.3‐, 3.2‐, and 5.4‐fold higher ATP secretion than cisplatin‐treated 4T1 cells, Pt‐CDs‐treated 4T1 cells after 589 nm laser irradiation, and Pt‐CDs@BSA‐treated 4T1 cells in the dark, respectively. CRT and HMGB1 expression were detected by immunofluorescent staining with an Alexa Fluor 488‐conjugated secondary antibody, which exhibited green FL for further CLSM observations. Pt‐CDs@BSA‐treated 4T1 cells after 589 nm laser irradiation presented significantly enhanced green fluorescence on the cell surface, indicating much stronger CRT expression (Figure 4I). HMGB1 is a danger signal that stimulates phagocytosis of dying tumor cells by dendritic cells (DCs). Strong green fluorescence from Alexa Fluor 488‐labeled HMGB1 was observed in the nucleus of the control‐, Pt‐CDs‐, and Pt‐CDs@BSA‐treated 4T1 cells in the dark, indicating low HMGB1 release (Figure 4I and Figure S22, Supporting Information). Weaker green fluorescence signals were observed in cisplatin‐treated 4T1 cells and Pt‐CDs‐treated 4T1 cells after 589 nm laser irradiation, indicating moderate HMGB1 release. In contrast, the green fluorescence signal from Pt‐CDs@BSA‐treated 4T1 cells after 589 nm laser irradiation was the weakest, indicating the highest HMGB1 release from the nucleus. All of these results demonstrated strong orange light‐induced ICD after treatment with Pt‐CDs@BSA, which is important to trigger an efficient antitumor immune response throughout the body.

### 2.6 In Vivo Biodistribution, Pharmacokinetics, and Tumor Accumulation

**Figure 5:**
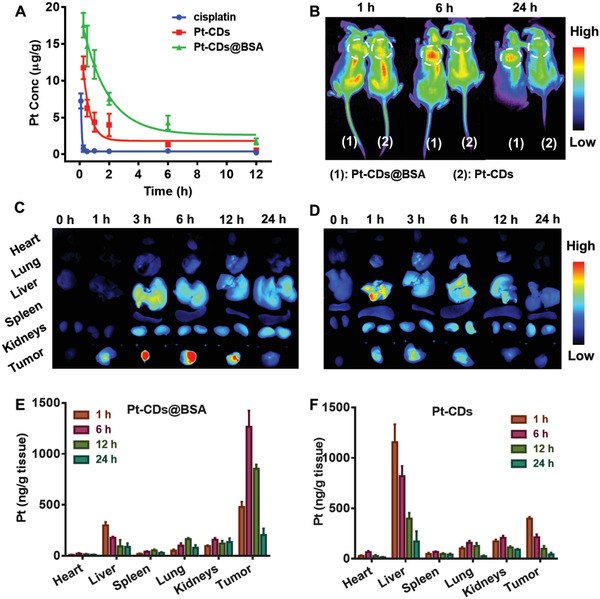
In vivo biodistribution, pharmacokinetics, and tumor accumulation of Pt(IV)‐coordinated carbon dots (Pt‐CDs) and Pt‐CDs@BSA. A) Pharmacokinetic profiles of cisplatin, Pt‐CDs, and Pt‐CDs@BSA in mice after tail vein injection. B) In vivo fluorescence images of mice bearing 4T1 tumors 1, 6, and 24 h after intravenous injection of Pt‐CDs and Pt‐CDs@BSA at a Pt dose of 2.5 mg kg^−1^. Ex vivo fluorescence images of the tumors and organs harvested from euthanized mice at different time points postinjection of C) Pt‐CDs@BSA and D) Pt‐CDs at a Pt dose of 2.5 mg kg^−1^. Pt biodistribution in the mice bearing 4T1 tumors 1, 6, 12, and 24 h after intravenous injection of E) Pt‐CDs@BSA and F) Pt‐CDs at a Pt dose of 2.5 mg kg^−1^. Data presented as mean ± S.D. (*n* = 3).

The blood circulation time of drugs is an important factor that influences the effects of antitumor treatment. Thereby, the pharmacokinetics of cisplatin, Pt‐CDs, and Pt‐CDs@BSA were evaluated by ICP‒MS. Based on the quantification of Pt in the blood after intravenous injection of these samples with a Pt dose of 2.5 mg kg^−1^, the half‐life time (*t*_1/2_) of the Pt‐CDs@BSA (≈2 h) was much longer than that of cisplatin (≈8 min) and Pt‐CDs (≈27 min) (**Figure** **5**A), suggesting that Pt‐CDs@BSA had a greater chance of tumor accumulation.

We further examined the in vivo biodistribution and tumor accumulation of Pt‐CDs and Pt‐CDs@BSA via FL imaging of tumor‐bearing mice to evaluate their feasibility for antitumor treatment. After intravenous injection of Pt‐CDs or Pt‐CDs@BSA at a Pt dose of 2.5 mg kg^−1^ into mice bearing 4T1 cell tumors, the whole body of each mouse gradually exhibited red FL (Figure 5B). Based on the changes in the red FL signals, the Pt‐CDs showed faint accumulation in the tumor area at 3 h postinjection and were rapidly cleared after 12 h. In contrast, Pt‐CDs@BSA gradually accumulated at the tumor area with a clear contrast in the red FL compared with other tissues in the long‐term time range of 3–6 h postinjection.

The major organs (heart, liver, spleen, lung, and kidneys) and tumors were excised and studied before and 1, 3, 6, 12, and 24 h after intravenous injection of Pt‐CDs or Pt‐CDs@BSA for ex vivo FL imaging to quantify their red FL intensities. As shown in Figure 5C, Pt‐CDs@BSA gradually accumulated in the tumor 3–6 h postinjection as determined by the significantly enhanced red FL in the tumor which was much higher than that in the other organs, which is consistent with the in vivo FL imaging results. In contrast, Pt‐CDs exhibited much weaker tumor accumulation with no significant red FL signal differences among the tumor, liver, and kidney throughout the observation period. At 24‐h postinjection of Pt‐CDs and Pt‐CDs@BSA, the FL from the tumors and other organs significantly decreased. The accumulation of Pt in the tumor and other major organs at 1, 6, 12, and 24 h postinjection was further determined by ICP‒MS. As shown in Figure 5E, in the Pt‐CDs@BSA‐treated mice, Pt gradually accumulated in the tumor and reached a maximum content at 6 h postinjection, which was much higher than that in the other organs throughout the observation period, agreeing well with the in vivo and ex vivo FL imaging results. In contrast, in the Pt‐CDs‐treated mice, the accumulation of Pt in the tumor was much weaker than that observed in the Pt‐CDs@BSA‐treated mice. The Pt accumulation in all major organs and tumors decreased to a low level in both the Pt‐CDs@BSA‐ and Pt‐CDs‐treated mice, indicating that most of the Pt‐CDs and Pt‐CDs@BSA can be cleared from the body within 24 h, which agreed well with the in vivo and ex vivo FL imaging data. These results clearly illustrated that Pt‐CDs@BSA with larger particle sizes exhibited a longer blood circulation time and higher tumor accumulation due to the significantly enhanced permeability and retention (EPR) effect, showing great potential for precise tumor therapy.

### 2.7 Orange Light‐Induced Tumor Eradication and ICD‐Induced Antitumor Inhibition

**Figure 6:**
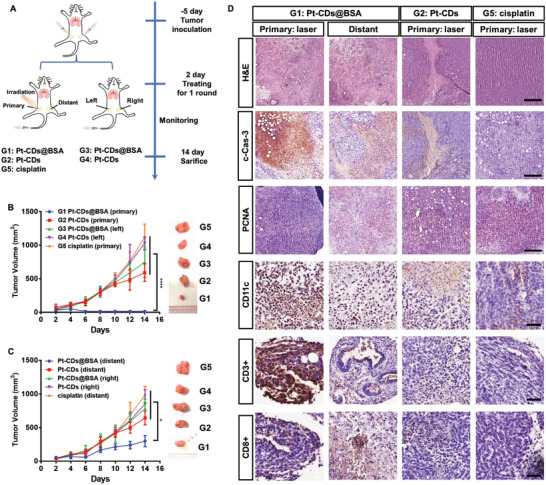
In vivo photoinduced antitumor effect of Pt‐CDs@BSA against 4T1 breast tumors. A) Treatment schedule for photoactivation combination therapy. B) Primary tumor and C) distant tumor growth curves of the 4T1 tumors in BALB/c mice intravenously injected once with the Pt‐based samples (each with 2.5 mg Pt kg^−1^): G1: Pt‐CDs@BSA with primary tumor laser irradiation; G2: Pt‐CDs with primary tumor laser irradiation; G3: Pt‐CDs@BSA; G4: Pt‐CDs; and G5: cisplatin with primary tumor laser irradiation. D) Hematoxylin and eosin (H&E), cleaved caspase 3 (c‐Cas‐3), and PCNA staining of the tumors after one round of treatment. Scale bar: 250 µm. CD3^+^, CD8^+^, and CD11c^+^ immunohistochemical (IHC) staining of tumors in each group. Scale bar: 50 µm. Data presented as mean ± S.D. (*n* = 5); *p*‐values are calculated using one‐way ANOVA, *p < 0.05, ^****^ *p* < 0.001).

In general, satisfactory tumor eradication and stimulation of antitumor immunity using Pt‐based drugs or prodrugs require several rounds of treatment, particularly in combination with immune checkpoint blockade therapy, which might potentially increase systemic toxicity, cause drug resistance issues, and bring about continuous pain to patients from treatment. Therefore, curing tumors by administering one dose or one round of treatment is highly appealing. Encouraged by its high tumor accumulation, excellent cancer cell killing capacity and strong ICD induction, one round of orange light‐induced tumor therapy with Pt‐CDs@BSA was carried out in a murine bilateral tumor model; namely, 4T1 bilateral tumors in BALB/c mice, as shown in **Figure** **6**A. Allograft bilateral tumors were established by implanting 4T1 cells (5 × 10^5^) into the left and right 4th mammary fat pads of normal female BALB/c mice. The left and right tumors were designated as the primary and distant tumors, respectively. When the primary tumor volume reached ≈50 mm^3^, these mice were randomized into five groups (G1–G5, *n* = 5 in each group) and received treatment (drugs were injected intravenously): (G1) Pt‐CDs@BSA followed by laser irradiation (Pt‐CDs@BSA + laser); (G2) Pt‐CDs followed by laser irradiation (Pt‐CD + laser); (G3) Pt‐CDs@BSA in the dark; (G4) Pt‐CDs in the dark; and (G5) cisplatin followed by laser irradiation (cisplatin + laser). The dose of Pt for each group was 2.5 mg Pt kg^−1^. For the groups undergoing laser irradiation, a 589 nm laser (0.5 W cm^−2^, 10 min) was placed on the primary tumors 6 h after injection, whereas the right distant tumors were kept in the dark. The mice in the nonirradiated Pt‐CDs and Pt‐CDs@BSA groups, tumor volume rapidly increased over time (Figure 6B). Among the irradiated groups, the growth of the primary tumors in the mice treated with Pt‐CDs was partially inhibited, while the growth of the primary tumors in the Pt‐CDs@BSA‐treated mice was significantly depressed and finally stopped (Figures S23 and S24, Supporting Information). It should be noted that one round of cisplatin treatment with the same dose of Pt and laser irradiation had no effect on the primary tumors. Along with the superior orange light‐induced antitumor efficacy against the primary tumors, the growth of the distant tumors was also clearly inhibited in the Pt‐CDs@BSA + laser group (Figure 6C). In addition, it was noted that these treatments did not cause obvious changes in the body weights of the mice in each group (Figure S25, Supporting Information), indicating their negligible systemic toxicity with the low dose of Pt.

On day 14, the tumors of all mice were surgically removed and weighed, followed by standard hematoxylin and eosin (H&E) and immunohistochemical (IHC) staining. The Pt‐CDs@BSA + laser group exhibited almost negligible primary tumors and much smaller distant tumors than the other groups, confirming their excellent antitumor efficacy. Analysis of the tumor tissues revealed a large area of apoptotic cells with apparent nuclear shrinkage and a loss of membrane integrity in the primary tumors of the Pt‐CDs@BSA + laser group (Figure 6D and Figure S26, Supporting Information). Notable cellular apoptosis was also observed in their distant tumors. In contrast, slight cellular apoptosis was observed in the primary tumors of the Pt‐CDs + laser group, while no clear cellular apoptosis was observed in tumors of G2–G5 mice. We further measured the protein levels of cleaved caspase 3 and PCNA by IHC staining to investigate the levels of apoptosis in tumors. The results showed that the primary tumors of the mice in the Pt‐CDs@BSA + laser group exhibited markedly increased cleaved caspase 3 levels and dramatically decreased PCNA levels, indicating the most significant cellular apoptosis. Based on the protein expression of cleaved caspase 3 and PCNA, the order of observed apoptosis from high to low was the primary tumors of the Pt‐CDs@BSA + laser group, the distant tumors of the Pt‐CDs@BSA + laser group, and the primary tumors of the Pt‐CDs + laser group; the tumors of the mice in G2–G5 did not exhibit obvious cellular apoptosis. It can thus be inferred that 589 nm laser irradiation of the primary tumor combined with Pt‐CDs@BSA treatment not only killed the primary tumor but also triggered an efficient antitumor response throughout the body to inhibit the growth of the distant tumor.

The antitumor immune responses were further analyzed by examining the characteristic protein expression levels of DC maturation (CD11c^+^), CD3^+^, and CD8^+^ T cells in the tumors (Figure 6D and Figure S27, Supporting Information). Mature DCs play a crucial role in antigen presentation to T lymphocytes, thus promoting intratumoral infiltration of CD8^+^ cytotoxic T lymphocytes (CTLs).^[^ ^18^, ^20^ ^]^ The primary tumors of the mice in the Pt‐CDs@BSA + laser group exhibited notably high CD11c^+^ and CD3^+^ expression levels, indicating high intratumoral infiltration of mature DCs and total T cells. It should be noted that the CD8^+^ expression in the primary tumors of the mice in the Pt‐CDs@BSA + laser group was not significant, which could be because this group had the highest number of killed cancer cells in the primary tumors. Enhanced CD11c^+^, CD3^+^, and CD8^+^ expression levels were also observed in the distant tumors of the Pt‐CDs@BSA + laser group, which were even higher than those of the primary tumors of the Pt‐CDs + laser group. In contrast, insignificant intratumoral infiltration of CD11c^+^, CD3^+^, and CD8^+^ expression was observed in the tumors of the mice in G2–G5, indicating that the ICD induced in the primary tumors by orange light after treatment with Pt‐CDs@BSA can trigger a robust anticancer immune response to achieve an abscopal antitumor effect after only one round of treatment.

### 2.8 Inhibition of Lung Metastasis by ICD Effects In Vivo

**Figure 7:**
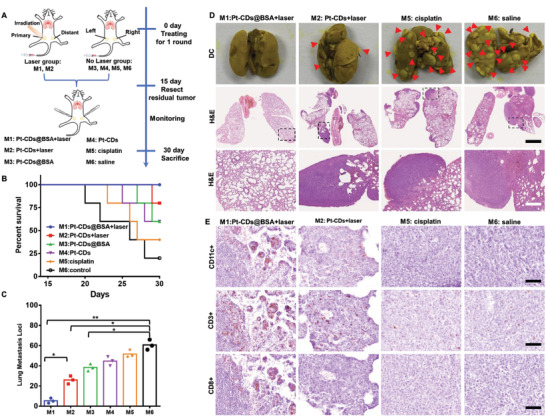
Antimetastatic effects in 4T1 tumor‐bearing BALB/c mice. A) Schematic illustration of lung metastasis inhibition via a systemic antitumor immune response. B) Survival rate profiles (*n*  =  5) of the mice with 4T1 lung metastases after different treatments. C) Metastatic lung nodule counting from mice intravenously injected with 4T1 tumor cells receiving different treatments. D) Digital photographs of lungs with metastatic nodules stained by Bouin's fluid and hematoxylin and eosin (H&E) staining of lung sections from 4T1‐tumor‐bearing mice treated with saline, cisplatin, Pt‐CDs + laser, and Pt‐CDs@BSA + laser. The red circles indicate metastatic nodules. Scale bars: the black scale bar is 2.5 mm, and the white scale bar is 1.0 mm. E) CD11c^+^, CD3^+^, and CD8^+^ immunohistochemical (IHC) staining of the lungs from mice in each group. Scale bar: 50 µm. Data presented as mean ± S.D. (*n*  =  5). Injection dose: 2.0 mg Pt kg^−1^. Statistical significance was calculated via a one‐way ANOVA test for the data. *p <0.05, ^**^ *p* <0.01.

Distant metastasis of breast cancer, particularly to the lung, is one of the great challenges in cancer therapy and often causes cancer patient death.^[^ ^21^ ^]^ Excitingly, one round of 589 nm laser treatment after Pt‐CDs@BSA had accumulated in tumors effectively prevented lung metastasis. After the above allograft bilateral 4T1 mouse tumor experiments, all of the tumors in groups M1–M5 and the control group (M6) were surgically removed on day 15, and construction of the survival curve continued (**Figure** **7**A). After day 22, continuous mouse death was observed in groups M2–M5, while all mice survived in group M1 (Figure 7B). On day 30, all mice were sacrificed, and their lungs were collected for further analysis. The lung metastatic nodules stained by Bouin's fluid were counted and photographed.

As shown in Figure 7C,D and Figure S28 (Supporting Information), obvious lung metastasis (indicated by red arrowheads) occurred in groups M2–M6, while no clear lung metastasis was observed in the Pt‐CDs@BSA + laser (M1) group. Our data indicated that the numbers of lung nodules diminished by approximately 57% and 91% in the Pt‐CDs + laser (M2) and Pt‐CDs@BSA + laser (M1) groups relative to the control group (M6), respectively (Figure 7C), indicating that pulmonary metastasis was effectively prevented after one round of 589 nm laser irradiation combined with Pt‐CDs@BSA treatment. The H&E results showed a dramatic decline in the number of metastatic lesions in the lungs in the Pt‐CDs@BSA + laser (M1) group, which was in sharp contrast to groups M2‐M6, in which large areas of metastatic lesions were observed in the lungs (Figure 7D and Figure S29, Supporting Information).

To verify the activation of the systemic immune response, DC maturation and T‐cell populations in lung tissues after different treatments were investigated. The number of CD11c^+^ DC cells significantly increased in the Pt‐CDs@BSA + laser group in comparison with the other groups (Figure 7E and Figure S30, Supporting Information). Consistently, the infiltration of CD8^+^ and CD3^+^ T cells in the lung tissues notably increased, indicating the increased proliferation and activation of effector CTLs to kill metastatic tumors.

**Figure 8:**
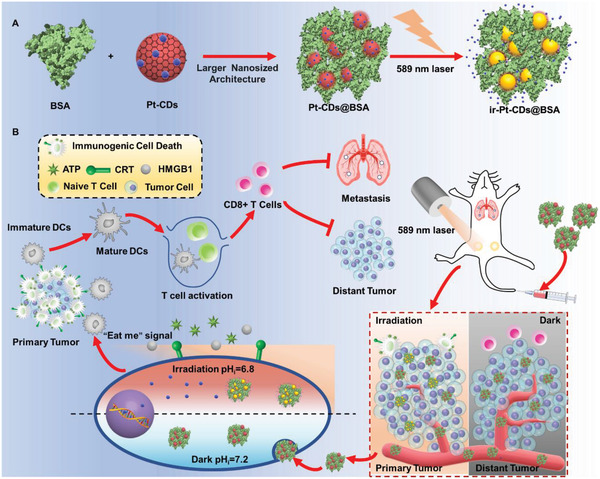
The proposed mechanism by which Pt‐CDs@BSA effectively induce immunogenic cell death (ICD) after orange light irradiation for precision antitumor therapy. A) Illustration of the bovine serum albumin (BSA) composite Pt‐CDs. B) A proposed mechanism of ICD effect induced by Pt‐CDs@BSA after orange light to eliminate metastatic tumors.

A possible mechanism for the excellent, precise, orange light‐induced antitumor therapeutic performance with Pt‐CDs@BSA is proposed in **Figure** **8**. Pt‐CDs@BSA exhibit enlarged particle sizes with much higher cellular uptake and tumor accumulation than Pt‐CDs and inherited the excellent orange light‐induced excellent cancer cell killing capacities of Pt‐CDs due to cytotoxic Pt(II) species release, hydroxy radical generation, and acidification. After a single intravenous injection of Pt‐CDs@BSA followed by 10 min of 589 nm laser irradiation, the orange‐light induced release of Pt(II) species, generation of hydroxy radicals, and intracellular acidification caused significant ICD of cancer cells. The CRT exposure, HMGB1 release, and ATP secretion caused by ICD recruit DCs to the treated primary tumor and promote the maturation of DCs to capture the tumor antigens from the cancer cell debris. These antigens are further presented to T cells to trigger antitumor responses throughout the body, inhibit abscopal tumors, and prevent tumor metastasis.

## 3 Conclusion

In summary, we developed, for the first time, solvothermally synthesized photoactivatable Pt(IV)‐coordinated CDs (Pt‐CDs) and their BSA complex (Pt‐CDs@BSA). Pt‐CDs@BSA exhibit enlarged particle sizes of 50–120 nm with much higher cellular uptake and tumor accumulation than pure Pt‐CDs. Pt(IV) is homogeneously distributed in the Pt‐CDs (Pt: 17.2 wt%), and their carbon cores display a significant visible absorption band to exhibit excellent photocatalytic properties, efficiently reducing Pt(IV) to Pt(II) and promoting hydroxy radical generation from water under orange light. This unprecedented system with orange light‐triggered ultrastrong cancer cell killing capacities due to efficient cytotoxic Pt(II) species release, hydroxy radical generation, and intracellular acidification causes much stronger ICD than cisplatin at the same Pt dose in vivo. After Pt‐CDs@BSA accumulation in the tumor following intravenous injection, one round of 10 min of 589 nm laser irradiation (0.5 W cm^−2^) not only killed the primary tumor but also inhibited distant tumor growth and lung metastasis, indicating superior antitumor and anti‐metastatic efficacy. These results demonstrate the new concept of building Pt(IV)‐enriched prodrug‐based photocatalytic CDs for precision cancer therapy.

## Conflict of Interest

The authors declare no conflict of interest.

## Supporting information

Supporting Information

## Data Availability

The data that support the findings of this study are available from the corresponding author upon reasonable request.
