# Correction to “Photocatalytic Pt(IV)‐Coordinated Carbon Dots for Precision Tumor Therapy”

**DOI:** 10.1002/advs.202518827

**Published:** 2025-10-05

**Authors:** D. Guo, J. H. Lei, D. Rong, T. Zhang, B. Zhang, Z. Tang, H.‐M. Shen, C.‐X. Deng, S. Qu

Adv. Sci. 2022, Dec. 9(36):2205106.

https://doi.org/10.1002/advs.202205106

In the originally published article, we found that the H&E image of the Distant tumor in the Pt‐CDs@BSA+laser group in Figure 6D was improperly used during the layout of the images. These corrections do not alter the overall analysis, interpretation, or conclusions of the study. The corrected image is presented below.

 
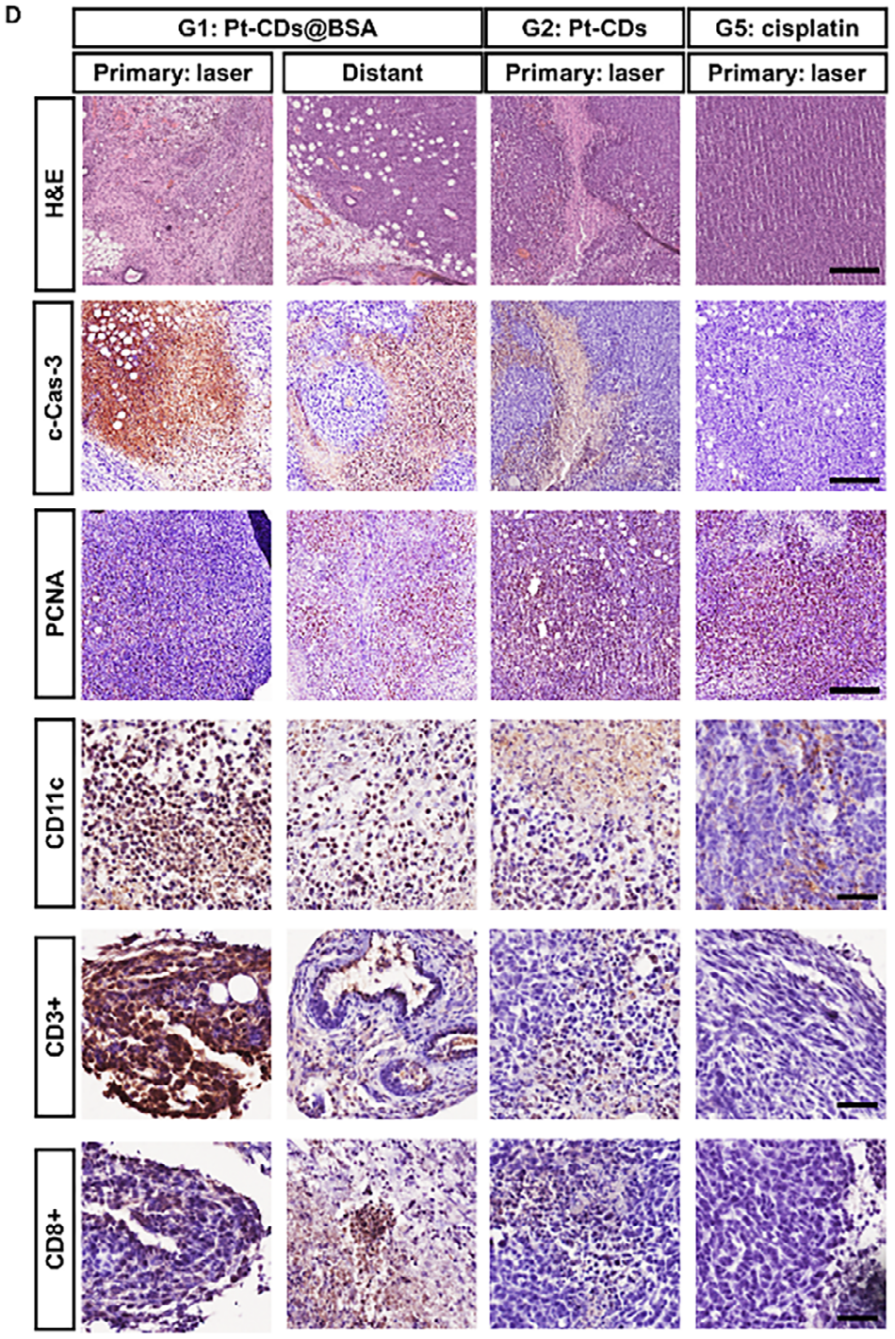


We apologize for this error.

