# Supplementary material for: Photocatalytic Pt(IV)‐Coordinated Carbon Dots for Precision Tumor Therapy
Source: Adv Sci (Weinh). 2022 Oct 28;9(36):2205106. doi: 10.1002/advs.202205106 (PMC9798972; doi:10.1002/advs.202205106)
Supplement: Supplementary file 1 — Supporting Information [file ADVS-9-2205106-s001.pdf]

ADVANCED SCIENCE
Open Access

Supporting Information

for *Adv. Sci.*, DOI 10.1002/advs.202205106

Photocatalytic Pt(IV)-Coordinated Carbon Dots for Precision Tumor Therapy

Dongbo Guo, Josh Haipeng Lei, Dade Rong, Tesen Zhang, Bohan Zhang, Zikang Tang, Han-Ming Shen, Chu-Xia Deng* and Songnan Qu**

Supporting Information

Photocatalytic Pt(IV)-Coordinated Carbon Dots for Precision Tumor Therapy

Dongbo Guo[#], Josh Haipeng Lei[#], Dade Rong[#], Teseng Zhang, Bohan Zhang, Zikang Tang, Han-Ming Shen, Chu-Xia Deng*, and Songnan Qu**

Dr. D. B. Guo, T.S. Zhang, B.H. Zhang, Prof. Z. K. Tang, Prof. S. N. Qu
Joint Key Laboratory of the Ministry of Education, Institute of Applied Physics and Materials Engineering, University of Macau, Taipa, Macau SAR, China.

*E-mail: songnanqu@um.edu.mo

Dr. J. H. Lei, D. D. Rong, Prof. H.-M. Shen, Prof. C.-X. Deng
Faculty of Health Sciences, University of Macau, Macau SAR, China.
*E-mail: hmshen@um.edu.mo, cxdeng@um.edu.mo

Dr. J. H. Lei, Prof. Z. K. Tang, Prof. H.-M. Shen, Prof. C.-X. Deng, Prof. S. N. Qu
MOE Frontier Science Centre for Precision Oncology, Cancer Center, Faculty of Health Sciences, University of Macau, Taipa, Macau SAR, China.

Prof. Z. K. Tang, Prof. S. N. Qu
Department of Physics and Chemistry, University of Macau, Taipa, Macau SAR, China.

Dr. D. B. Guo
School of Biomedical Engineering, State Key Laboratory of Marine Resource Utilization in South China Sea, Hainan University, Haikou 570228, China.

Keywords: carbon dots; Pt(IV) enriched prodrug; photocatalyst; orange light; immunogenetic cell death; tumor therapy

#These authors contributed equally.

Materials and Methods

Materials. All chemicals were of analytical grade and used without further purification if not indicated otherwise. Cisplatin, hydrogen peroxide, acetone o-phenylendiamine, p-phenylendiamine, 3-(4,5-dimethylthiazol-2-yl)-2,5-diphenylte-trazolium bromide (MTT), and bovine serum albumin (BSA) were purchased from Aladdin. Ferrostatin-1, propidium iodide, Z-VAD, and N-acetylcysteine were purchased from MCE (MedChemExpress). Live/Dead Cell Double Staining Kit, sodium hydrosulfite, anhydrous dimethylformamide (DMF), lysozyme, pepsin, trypsin, and other chemicals were purchased from Sigma-Aldrich. LysoTracker Green, ATP assay kit and 2',7'-bis-(2-carboxyethyl)-5-(and-6)-carboxyfluorescein acetoxymethyl ester (BCECF-AM) was purchased from Beyotime. HMGB1 Polyclonal Antibody, Calreticulin Polyclonal Antibody, and Donkey anti-Rabbit IgG (H+L) Highly Cross-Adsorbed Secondary Antibody, Alexa Fluor™ Plus 488 were purchased from ThermoFisher Scientific. Cleaved Caspase-3 (Asp175) Antibody, CD3e Monoclonal Antibody (145-2C11), CD8α (D8A8Y) Rabbit mAb, CD11c (D1V9Y) Rabbit mAb, anti-phospho-H2A.X (Ser139), and F4/80 monoclonal antibody were purchased from Cell Signaling Technology (CST). Anti-PCNA antibody was purchased from Abcam.

Characterizations. Transmission electron microscopy (TEM) was performed on an FEI Tecnai-G2-F20 transmission electron microscope (200 kV). An Inca X-Max instrument was used for perform the EDS and elemental mapping. The XPS analyses were conducted on an ESCALAB 250Xi photoelectron spectrometer using Mo as the exciting source (Thermo Fisher Scientific). A Shimadzu UV-2600 spectrophotometer was used for collected the UV-visible absorption spectra, and the emission spectra were acquired from a Horiba Jobin Yvon Fluorolog-3 spectrometer (Xenon lamp excitation). The photoluminescence quantum yield of all nanomaterials was collected at room temperature on an Edinburgh FS5 spectrophotometer. The fluorescence imaging of cell was collected from an Olympus FV1000 confocal laser scanning microscope. The laser (689 nm) was generated from cnilaser MD-655NM-HS-2W-16060512. The pH values of the CD aqueous solutions were adjusted by precise pH Meter (PHS-25, China). The Pt analyses was conducted on iCAP Qs Inductive Coupled Plasma Mass Spectrometer (ICP-MS, Thermo Fisher Scientific). IHC images were acquired with an Olympus IX83 inverted microscope (Olympus Corp., Tokyo, Japan).

Drug release of Pt-CDs and Pt-CDs@BSA. 10 mg of lyophilized Pt-CDs (Pt content 17.6 wt%) was dissolved in phosphate buffered saline (0.1 M PBS, pH =7.4) in dialysis bags, which were put into glass bottles with 0.1 M PBS, or 10 mM GSH, respectively. For light

irradiation group, a 589-nm light was placed on 5 min every 30 min for five times. The irradiated dialysis bag was then put in phosphate buffered saline (0.1 M PBS, pH =7.4). At timed intervals, 2 mL of external buffer was withdrawn from the reservoir and analyzed by ICP-MS, and then 2 mL of the fresh buffer was added to keep the volume of the release medium invariable. All measurements were performed in triplicate, and the releasing content was calculated by formula 1:

Releasing content (%) = (amount of drug in the releasing medium)/ (amount of drug loaded into nanogels) (1)

The pH regulation of photoactivatable Pt-CDs. 10 mg of lyophilized Pt-CDs (Pt content 17.6 wt%) was dissolved in H_2O. Different light sources were placed on the solutions by various light conditions. At timed intervals, the pH in solution was measured using a microprobe-based pH system (Micro pH Electrode, Thermo Fisher).

Cell uptake of Pt-CDs. 4T1 cells were obtained from Chinese Academy of Sciences Cell Bank (Shanghai, China). 4T1 cells were maintained in RPMI 1640 medium, supplemented with 10% FBS (v/v), 100 mg/mL streptomycin and 100 U/mL penicillin at 37 °C in a humidified atmosphere with 5% CO_2. 4T1 cells (1.2 $\times 10^6$ cells/well) were seeded in 6-well plates. Then, Pt-CDs and Pt-CDs@BSA (10 μM Pt) were further added to the medium for 1, 2, 4, and 6 h incubation. Then, the cells were washed 3 times with PBS, and cell nuclei were stained with DAPI (Beyotime Biotech, China) in order to identify the drug location inside cells. Images of cells were observed on an Olympus FV1000 confocal laser scanning microscope (Olympus FV1000, Japan) imaging system. Pt-CDs were excited at 488, and 543 nm, respectively.

For Pt content of cell internalization, Pt-CDs, and Pt-CDs@BSA were added to the medium for 1, 2, 4, 6, 12, and 24 h. Then, the cells were washed 3 times with PBS, treated with trypsin, centrifuged, and finally dispersed in 0.5 mL of PBS to measure Pt content in the samples.

Potential biotoxicity, uptake of Pt-CDs. Cytotoxicity profiles of cisplatin, Pt-CDs, and Pt-CDs@BSA against 4T1 cells were evaluated by the MTT assay. Cells were seeded on a 96 well plate (6000 cells per well) in 200 µL RPMI1640 incubated with the cisplatin, Pt-CDs, and Pt-CDs@BSA for 24 h at 37 °C. The cells were then treated with 200 µL fresh medium containing MTT (0.8 mg/mL) and incubated for 3 h at 37 °C. The medium was removed, 200 µL of DMSO was added to the cells, and the absorbance of the purple formazan was recorded at 570 nm using a Byonoy multi-detection microplate plate reader. Each experiment was performed in triplicate for each cell line.

Subcellular Localization. 4T1 cells were seeded at a density of 1 × 10^5 cells/well in a 24-well plate over glass coverslips and incubated at 37 °C under 5% CO_2 overnight. After being incubated with Pt-CDs@BSA ([Pt] = 10 μM) for 4 h at 37 °C, the culture medium was carefully aspirated, washed with DPBS three times, and fixed with 1 mL of 4% paraformaldehyde for 15 min, followed by staining with Lyso Tracker Green for additional 0.5 h at 37 °C in dark. After the extracellular fluorescence was quenched, cells were washed twice with PBS and fixed with 4% paraformaldehyde for 30 min. Then, cells were mounted on glass slides and visualized using confocal microscope (FluoView TM FV1000, Olympus, Japan).

Intracellular pH regulation of Pt-CDs and Pt-CDs@BSA. 2′,7′-Bis-(2-carboxyethyl)-5-(and-6)-carboxyfluorescein, acetoxymethyl ester (BCECF-AM) was used as a measure of intracellular pH value. Intracellular pH was determined from the pH-dependent ratio of emission intensity (535 nm) when the dye is excited at 488 nm (pH-dependent) versus the emission intensity when excited at its isobestic point of 440 nm (non-pH dependent) using a microplate reader (Absorbance 96, Byonoy GmbH). As previously described, a standard curve was calibrated with BCECF-AM-loaded dye over multiple pH values, and the linear range is 6.0 to 8.0 pH. Cells were incubated with 1 μM BCECF-AM for 20 minutes at 37°C and then washed three times with PBS prior to pH measurements.

Extracellular pH regulation of Pt-CDs and Pt-CDs@BSA. Using 4T1 cells grown in 35-mm^2 dishes, extracellular pH was measured using a microprobe-based pH system (Micro pH Electrode, Thermo Fisher). The electrode was calibrated with pH standards and placed in the media of the 35-mm^2 dishes containing the cells. pH measurements were made using an Accumet Ab150 pH meter (Fisher Scientific).

Synthesis of Pt-based Pt-CDs and BSA trapped with Pt-CDs. First, cisplatin (2.0 g, 6.6 ×10^5 mol) was suspended in water (50 mL) and an excess of H_2O_2 (30% w/v, 70 mL, 6.0 × 10^4 mol) was added. The mixture was stirred for 6 h at 50 °C and a pale-yellow powder (oxoplatin) resulted. Second, 15 mg of o-phenylendiamine and 15 mg of oxoplatin were dissolved in 30 mL DMF, heated at the temperature of 200 °C for 6 h under solvothermal conditions in a reaction autoclave, and cooled to room temperature. Red solution was acquired and then dialyzed against DI water for several days using a 1,000 MWCO dialysis membrane, before being freeze dried for subsequent studies. Finally, Pt-CDs with dark powder was obtained. Then, 0.5 mg of Pt-CDs were dissolved into 0.5-10 mg/mL BSA solution to prepare BSA trapped with Pt-CDs (Pt-CDs@BSA) for 30 min under 30-50 ^oC.

Photoactivation of Pt-CDs. 10 mg of lyophilized Pt-CDs was dissolved in H_2O or DMF. The FL and UV-vis absorption of Pt-CDs was performed by Edinburgh FS5 spectrophotometer and Shimadzu UV-2600 spectrophotometer. Then, the lyophilized Pt-CDs was dissolved in H_2O in dialysis bags, which was put in glass bottles with water. A 589 nm light was utilized to irradiate at a power of 0.5 W/cm^2 for 30 min. The solutions outer and inner the dialysis bags were collected and lyophilized, respectively. The solid powders were examined by XPS and ^{1}H NMR.

Western Blot. 4T1 cells were seeded in 6 wells plate. After treatment, cells were scraped off in lysis buffer (containing 1% PPI and 1‰ DDT) after washed twice with cold PBS. Protein concentrations were measured through BCA assay (Bio-rad, USA). The protein was separated on a sodium dodecyl sulfate-polyacrylamide gel electrophoresis, transferred to PVDF membranes (Millipore, USA), and blocked with 5% non-fat dry milk in TBST. After washing three times with TBST, the following primary antibodies dissolved in antibody buffer (Keygentec, China) were used: anti-phospho-H2A.X (Ser139) (9718S, Cell Signaling Technology, USA) and anti-GAPDH (ab8245, abcam, USA). After the secondary antibody incubation, the membrane was washed three times with TBST and exposed with ECL (Millipore, USA).

Propidium Iodide (PI) exclusion assay. 4T1 cells were seeded in 12 wells plate. After treatment, both suspended and sticked cells were collected and washed twice with cold PBS. Cells were resuspended with 300 μL cold PBS containing 5 μg/mL PI (Sigma, USA) and subjected to flow cytometry for cell viability detection. Twenty thousand cells were examined, and the data were analyzed with FlowJo vX.07 software.

ROS detection. 4T1 cells were seeded in 12 wells plate. After designed treatment, CellROX Oxidative Stress Reagents (C10444, Thermo Fisher Scientific, USA) were added into the cells and incubated for 30 min. After incubation, cells were collected and washed third with cold PBS. Cells were resuspended with 300 μL cold PBS and subjected to flow cytometry for ROS features detection. Twenty thousand cells were examined, and the data were analyzed with FlowJo vX.07 software.

***In vivo* antitumor and antimetastasis effect.** In 4T1 cell-derived orthotopic mammary tumor mouse model, 4T1 cells were inoculated into fourth mammary fat pads of BALB/c mice. Primary and distant tumors were inoculated on the left and right side, respectively. After the tumor volume reached ~80 mm^3, mice were randomly divided into five groups ($n = 5$ per group): cisplatin, Pt-CDs, Pt-CDs@BSA, Pt-CDs+laser, and Pt-CDs@BSA+laser. After

inhaled of 2% isoflurane, the mice were anesthetized. 0.2 mL of free drug, Pt-CDs or Pt-CDs@BSA were injected via the lateral vein at 2 Pt mg/kg. Mice were treated via intravenous injection only one time and irradiated via 589 nm light (0.5 W cm^{-2}, 10 min) after 6 h of post injection. Then, tumor bearing mice were sacrificed on day 14 after initial drug treatment and various organs/tissues including liver, kidney, heart, lung, and primary/distant tumor were harvested, fixed, and stained with corresponding antibodies according to previously described methods. Images were acquired with an Olympus IX83 inverted microscope (Olympus Corp., Tokyo, Japan). The tumor volume was calculated as following: V = ((tumor length) × (tumor width)^2)/2. Mice body weight, mortality, and clinical status were carefully recorded. At the end of the experiment, the primary and distant tumors were collected for weighting. To evaluate the antimetastasis eff ect, all the one round treatment of tumor mice were resected the residual tumor on the day 14. The mice were sacrificed on day 30 after initial drug treatment. The lung tissues were then harvested and incubated immediately in Bouin's fluid. The number of metastatic nodules was counted by photographing. The survival time of the five mice in each group was monitored and recorded. The lung tissues were fixed with paraformaldehyde (4% v/v) and stained with CD3, CD8, and CD11c antibodies for immunohistochemical analysis.

Induction of immunologic cell death (ICD) *in vitro*. To determine ICD of the tumor cells, surface expression of calreticulin (CRT), extracellular release of HMGB1 and ATP secretion were examined *in vitro*. For immunofluorescence detection of CRT expression, 4T1 cells were seeded and cultured in 6-well plate (5 × 10^5 cells/well) for 24 h. Then, the cells were treated with 10 μM Pt of cisplatin, Pt-CDs, or Pt-CDs@BSA for 6 h. After refreshed by medium, the cells in light groups were irradiated by light (589 nm, 0.5 W/cm^2) for 10 min. The cells were then cultured in the dark for another 2 h. Subsequently, the cells were then washed twice with PBS, fixed with 4% paraformaldehyde, and permeabilized using 0.1% triton-100 for 10 min. Then, the cells incubated with anti-CRT antibody or anti-HMGB1 for 30 min, and then incubated with Alexa Fluor 488-conjugated secondary antibody for additional 30 min. The cells were then stained with DAPI for 20 min and observed using CLSM.

The ATP assay kit was utilized to examinate the extracellular secretion of ATP. In brief, 4T1 cells were seeded into 6-well plate at the density of 5 × 10^5 cells/well. The cell culture medium was refreshed 24 h later, and the cells were treated with various vesicle formulations for 6 h. The cell culture medium was refreshed. For the irradiation group, the cells were

irradiated with 589 nm laser (10 min, 0.5 W/cm^2). Then, the cells were cultured for another 2 h. Afterwards, the supernatants were collected, and the extracellular ATP content was measured with an ATP Assay Kit according to the manufacturer's instruction.

Pharmacokinetic studies. BALB/c mice (~20 g) were chosen to study the pharmacokinetics of cisplatin, Pt-CDs, and Pt-CDs@BSA. Mice were randomly divided into three groups (n = 3). The free cisplatin, Pt-CDs, and Pt-CDs@BSA solutions were intravenously injected via tail vein at the same Pt doses of 1 mg Pt/kg. The blood samples (0.5 mL) were taken from the eye socket at the 15 min, 30 min, 1 h, 2 h, 8 h, and 12 h time points after injection. Blood was treated with 2 mL 65% HNO_3 (14.4 mol/L), 2 mL 40% HF (22.5 mol/L), and 1 mL 72% $HClO_4$ (12.4 mol/L) to extract prodrug and heated until the solution was dried completely. Then the products were dissolved in 2 mL 1% HNO_3 and filtered, followed by the analysis using ICP-MS.

Animal Models. Female BALB/c mice (20 ± 1 g) and female nude mice (20 ± 1 g) were provided by Faculty of Health, University of Macau. All animal experiments were conducted according to the animal research guidelines provided by the Animal Care and Use Committee at the University of Macau.

***In vivo* biodistribution of Pt-CDs and Pt-CDs@BSA.** In 4T1 cell-derived orthotopic breast tumor mice model, 4T1 cells were inoculated into two mammary fat pads of Balb/c mice. On day 14 after tumor inoculation, the mice were randomly divided into 8 groups (n = 3) and intravenously injected with Pt-CDs and Pt-CDs@BSA (at an equivalent Pt dose of 2.5 mg/kg). After 1, 6, 12, and 24 h, mice were sacrificed immediately. Major organs (heart, liver, spleen, lung, and kidneys) and orthotopic tumors were harvested, and the weight of all organs was recorded. The Pt content of tissues and tumors were performed by ICP-MS.

Live Animals Imaging and Tissue Distribution of Pt-CDs and Pt-CDs@BSA. The 4T1 tumor models of the nude mice were generated by fat pads injection of 4T1 cell in 100 μL (5 × 10^5 cells) into the dorsal area of each 4T1 mouse. Mice bearing 4T1 orthotopic tumors were intravenous injected with Pt-CDs and Pt-CDs@BSA through tail vein. Primary tumors and various organs were collected and subjected to fluorescent imaging on an IVIS Spectrum Imaging System (Caliper Life Sciences Inc., USA) at different time points (n=3 at each point).

Histopathological evaluation. For histological analysis, the organs (heart, liver, spleen, lung, and kidney) were fixed in 10% formalin, then embedded in paraffin. Slices of these organs

from the mice were stained with Hematoxylin & Eosin (H&E) and Immunohistochemistry (IHC). The histological sections were imaged by an optical microscope.

Statistical analysis. Data are presented as mean ± S.D. Statistical significance of differences among groups were performed by Student's t-test. The differences between experimental groups and control groups were considered statistically significant for P value <0.05.

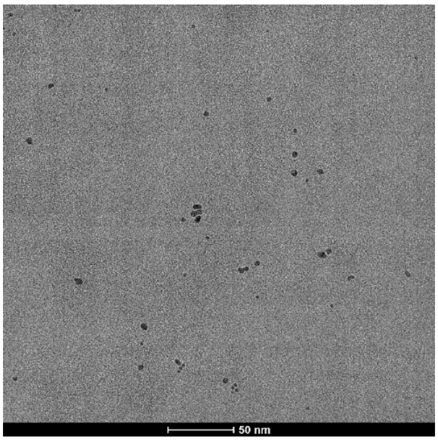

Fig. S1. TEM image of the Pt-CDs in large area.

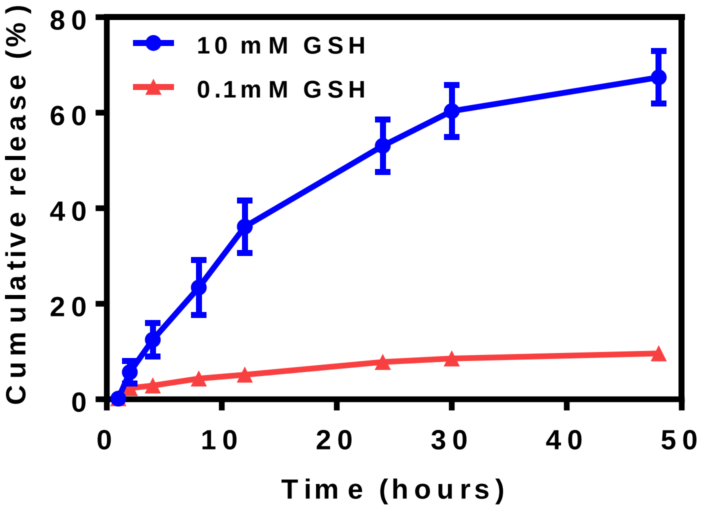

Fig. S2. Pt species content release profiles from Pt-CDs at different concentrations of GSH aqueous solutions.

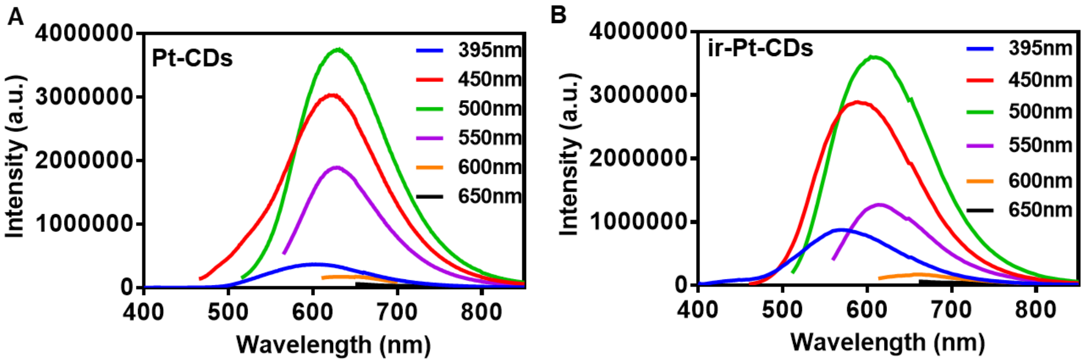

Fig. S3. The fluorescence spectra of (A) Pt-CDs and (B) ir-Pt-CDs in DMF.

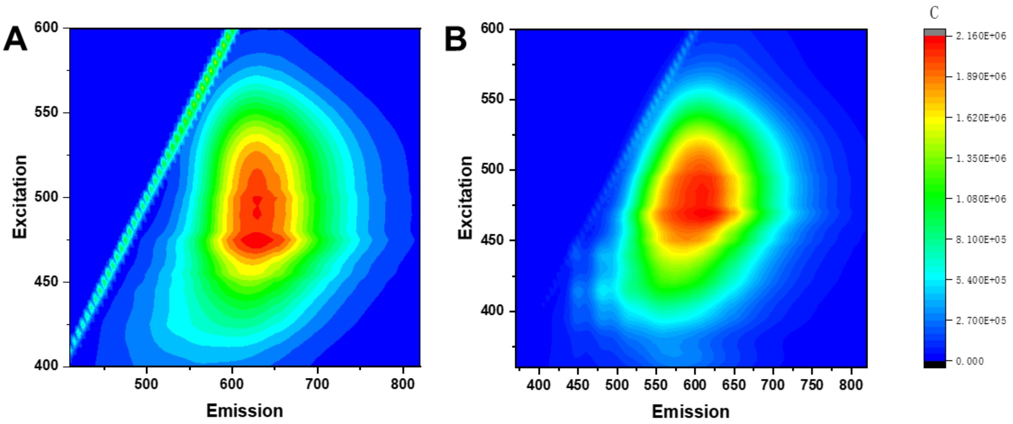

Fig. S4. Excitation-emission maps of (A) Pt-CDs and (B) ir-Pt-CDs in DMF.

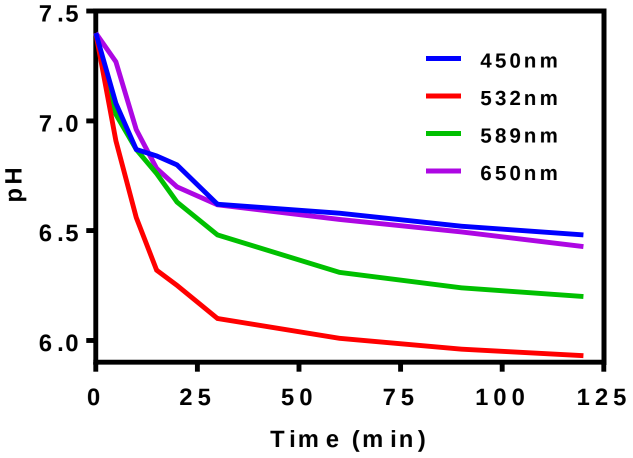

Fig. S5. The pH value variation curve of 0.5 mg/mL of Pt-CDs under 450, 532, 589, and 650 nm laser irradiation (10 min, 0.5 W/cm^2).

Table S1. The atomic ratio of C, N, O, Cl, and Pt in Pt-CDs, ir-Pt-CDs, and released species

	C(%)	N(%)	O(%)	Cl(%)	Pt(%)	Pt(wt%)
Pt-CDs	70.8	10.2	14.9	2.7	1.4	17.2
ir-Pt-CDs	69.2	9.8	19.4	1.2	0.4	6.0
Released species	N/A	33.8	6.7	36.8	22.7	7.1

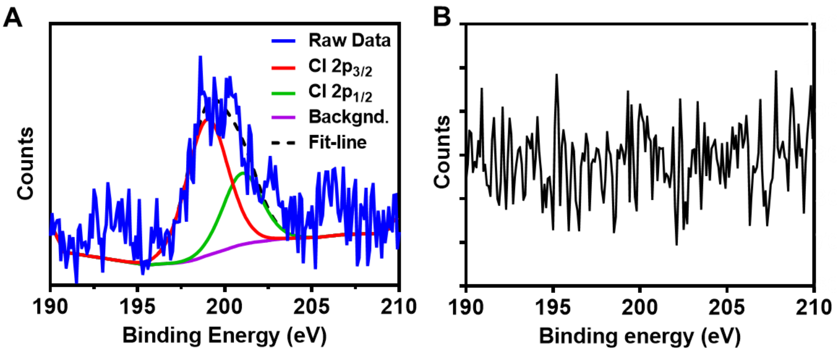

Fig. S6. High-resolution Cl2p spectra of (A) Pt-CDs and (B) ir-Pt-CDs.

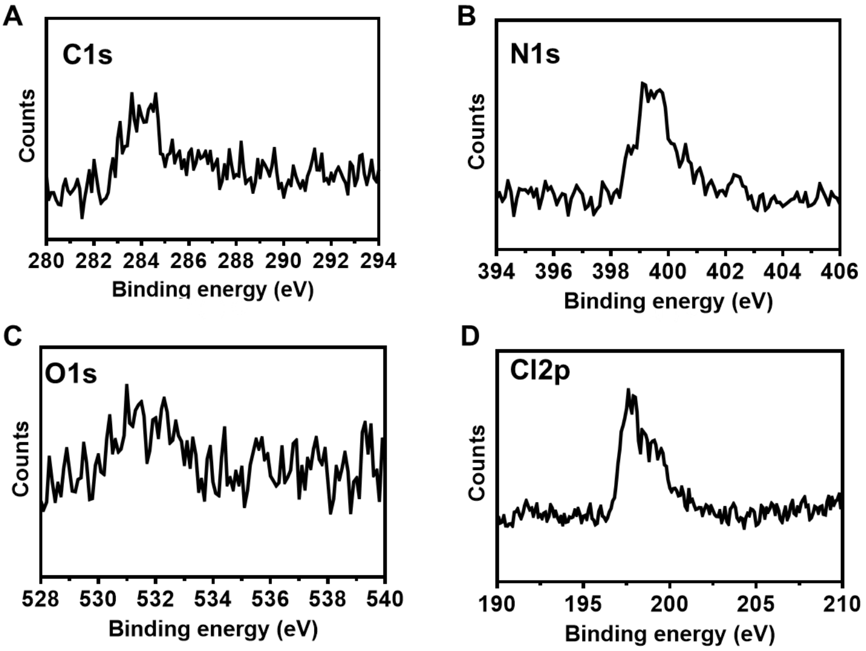

Fig. S7. High-resolution (A) C1s, (B) N1s, (C) O1s, and (D) Cl2p spectra of the released species from Pt-CDs with 30 min 589 nm laser irradiation (0.5 W/cm^2).

Table S2. Curving fitting results for XPS spectra of C 1s for Pt-CDs and ir-Pt-CDs

	Pt-CDs(%)	ir-Pt-CDs (%)
C=C	47.3	37.6
C-N	30.7	23.0
C-O	14.3	14.6
O=C-O	7.8	24.8

Table S3. Curving fitting results for XPS spectra of N 1s for Pt-CDs and ir-Pt-CDs

	Pt-CDs(%)	ir-Pt-CDs (%)
pyridinic	22.0	35.7
graphitic	37.5	41.3
$(NH_3)_2PtCl_2$	23.6	12.4
$C\text{-}NH_2$	16.9	10.6

Table S4. Curving fitting results for XPS spectra of O 1s for Pt-CDs and ir-Pt-CDs

	Pt-CDs(%)	ir-Pt-CDs (%)
C=O/Pt-O	42.3	18.9
C-O/C-O-C	43.3	44.6
O=C-O	14.5	36.6

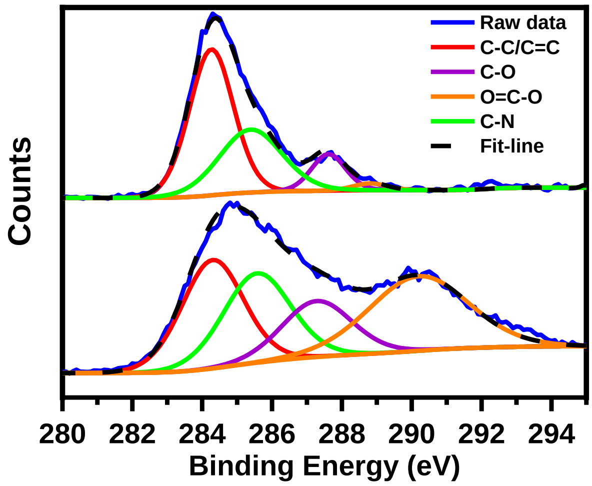

Fig. S8. High-resolution C1s spectra of Pt-CDs and ir-Pt-CDs.

Table S5. The chemical shift and integrations of protons of $Pt(NH_3)_2$ and COOH by 1H NMR spectra of Pt-CDs and ir-Pt-CDs in DMSO-d_6.

	Chemical Shift (ppm)	Integrations of protons		$\Delta_{Integrations}$
		Pt-CDs	ir-Pt-CDs	
$Pt(NH_3)_2$	6.35	130.51	72.45	58.06
$(COOH)_2$	10.21	17.16	36.26	19.1
$R\text{-}CH_3$	1.24	1000	1000	N.A.

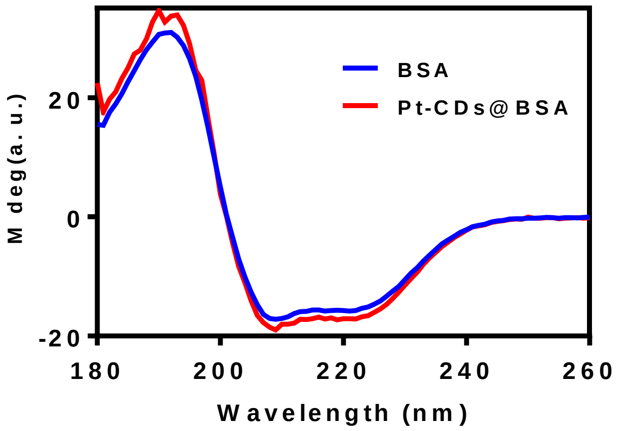

Fig. S9. Circular dichroism spectra of BSA and Pt-CDs@BSA

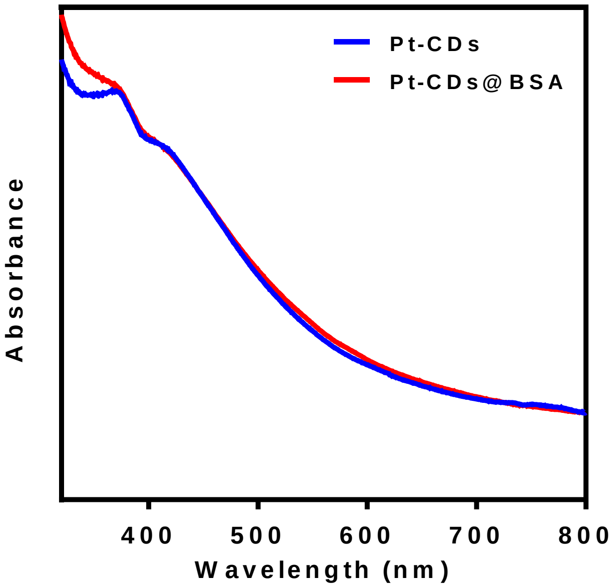

Fig. S10. UV/Vis absorption spectra of Pt-CDs and Pt-CDs@BSA in H_2O.

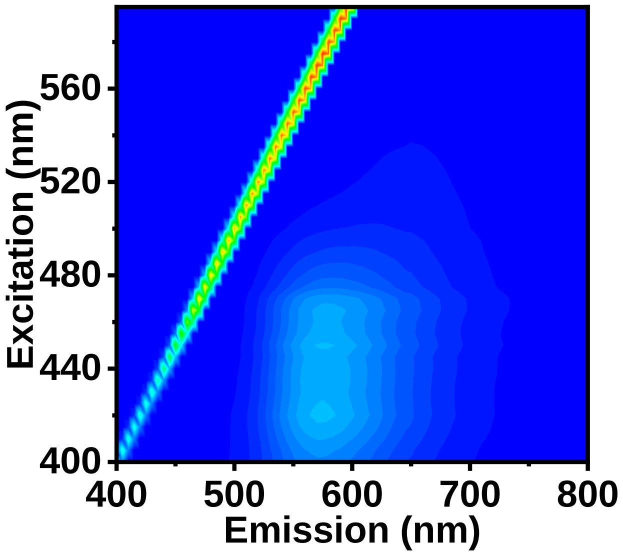

Fig. S11. Excitation-emission map of the Pt-CDs in H_2O.

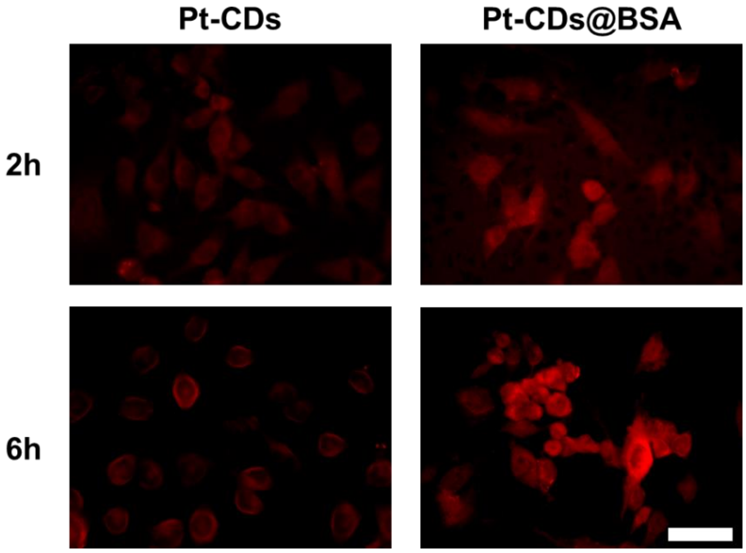

Fig. S12. Fluorescence images of 4T1 cells treated with Pt-CDs and Pt-CDs@BSA for 2 h and 6 h at a Pt content of 10 μM. Scale bar=20 μm.

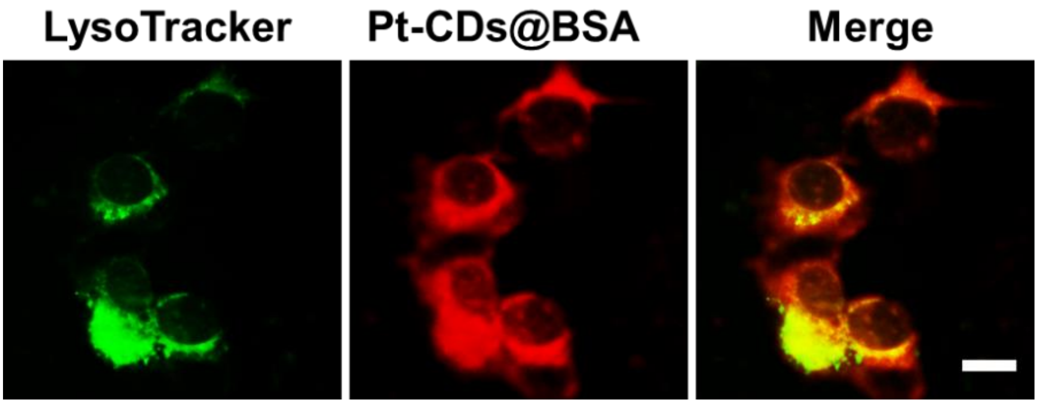

Fig. S13. Fluorescence images of 4TI cells after culturing with Pt-CDs@BSA for 4 h. Lysosome were stained by LysoTracker Green, respectively. (Scale bar: 5 μm).

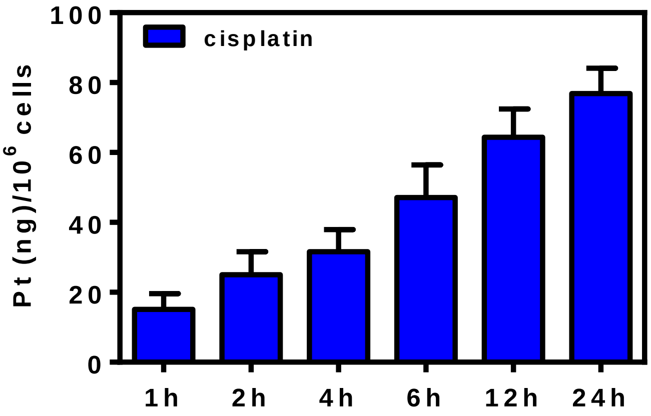

Fig. S14. Cellular uptake of cisplatin by 4T1 cells after 1-24 h of incubation at 37 ℃. The amount of Pt per cell was determined by ICP–MS.

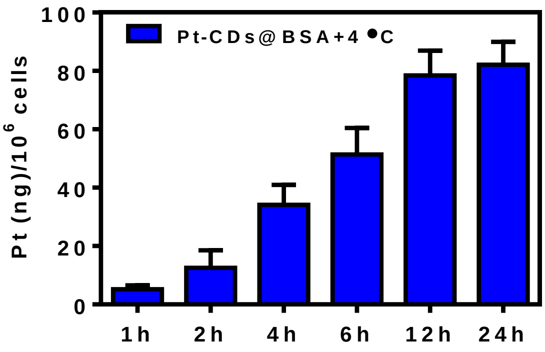

Fig. S15. Cellular uptake of Pt-CDs@BSA by 4T1 cells after 1-24 h of incubation at 4 ℃. The amount of Pt per cell was determined by ICP–MS.

Table S6. Internalized amounts of cisplatin, Pt-CDs, and Pt-CDs@BSA by 4T1 cells after 1-24 h of incubation at 37 ℃, and PBS at 4 ℃. The amount of Pt per cell was determined by ICP–MS.

	cisplatin ng/10^6 cells	Pt-CDs ng/10^6 cells	Pt-CDs@BSA ng/10^6 cells	Pt-CDs@BSA 4 ℃ ng/10^6 cells
1h	15.1±5.0	8.7±1.3	15.7±4.8	5.2±1.3
6h	47.1±10.0	8.5±3.4	167.2±19.4	51.4±9.2
12h	64.3±8.9	7.6±0.9	253.2±25	78.4±5.8

Table S7. The cytotoxicity of different complexes against 4T1 cells. The IC_{50} value was determined by the MTT assay.

Complex	Irradiation	IC_{50} (μM)	PI[a]
cisplatin	In the dark	42.5±5.1	1.1
	Under irradiation	39.6±4.8	
Pt-CDs	In the dark	389±18	9.1
	Under irradiation	42.8±4.9	
Pt-CDs@BSA	In the dark	322±16	31
	Under irradiation	10.2±2.5	

a Phototoxic index (PI): the IC_{50} of the dark group treated with compelx/the IC_{50} of the irradiation group treated with complex.

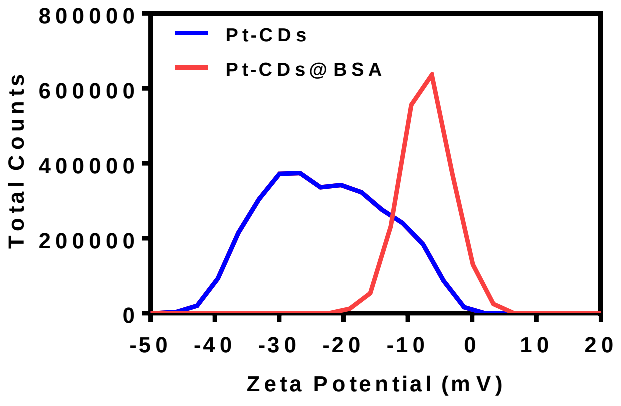

Fig. S16. Zeta potential of Pt-CDs and Pt-CDs@BSA in aqueous solutions.

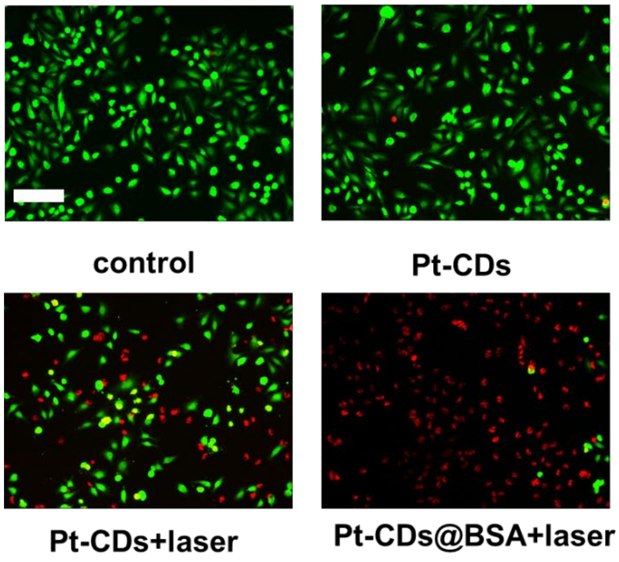

Fig. S17. Live/dead viability assay were performed for 4T1 cells with control, Pt-CDs, Pt-CDs+laser, and Pt-CDs@BSA+laser (589 nm, 0.5 W/cm^2, 10 min, 10 μM Pt). Scale bar: 50 μm.

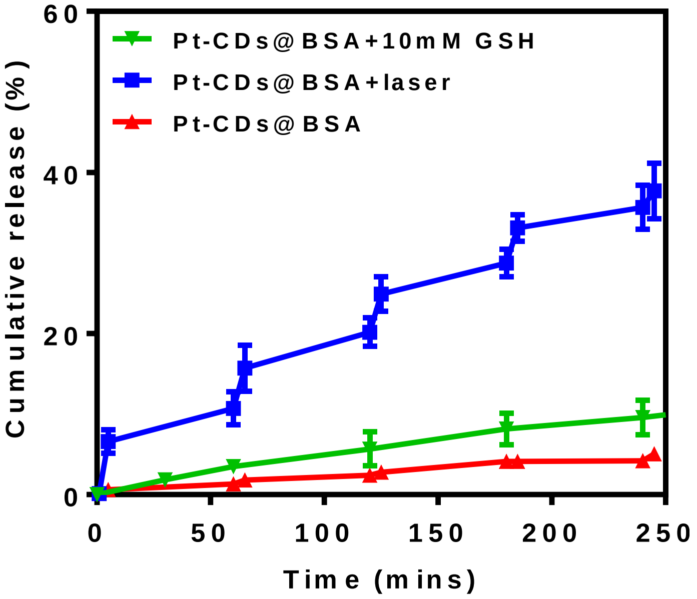

Fig. S18. Pt release profiles from 0.25 mg/mL Pt-CDs@BSA aqueous solutions with 10 mM GSH with or without 589 nm laser irradiation at 0.5 W/cm^2. The time for each laser irradiation was 5 min.

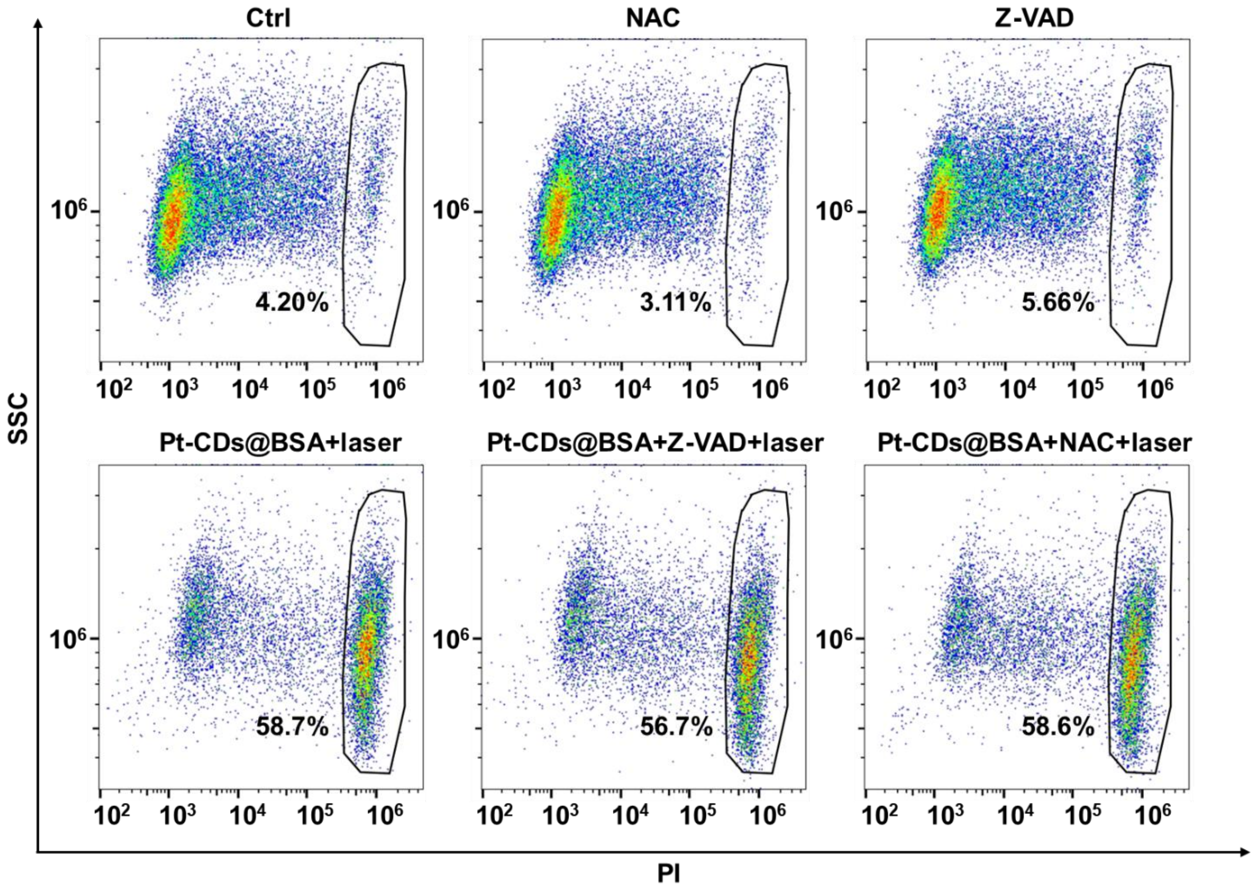

Fig. S19. PI exclusion assay of 4T1 cells treated with Z-VAD, NAC, Pt-CDs@BSA+laser, Pt-CDs@BSA+Z-VAD+laser, and Pt-CDs@BSA+ Fer-1+laser, [Z-VAD] = [NAC] = 10 μM.

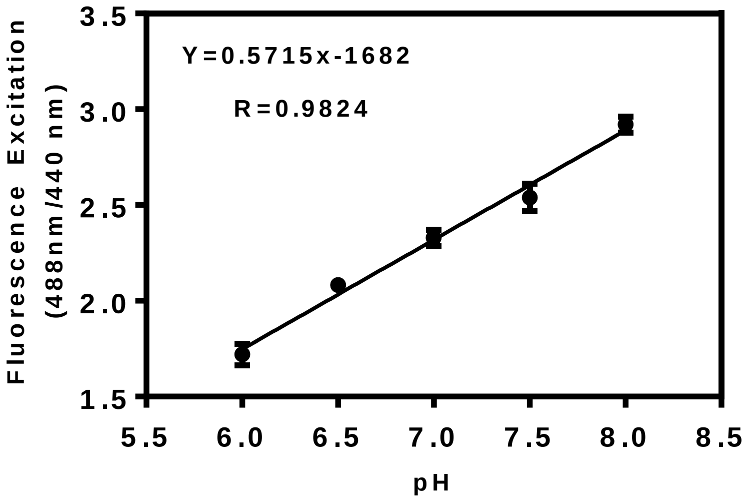

Fig. S20. Fluorescence intensity ratios vs pH for measuring intracellular pH. Standard curve of fluorescence intensity ratios of BCECF-AM vs pH. The fluorescence emission of 535 nm

was measured at excitations at 488 and 440 nm and was found to be linear between 6.0 and 8.0 pH.

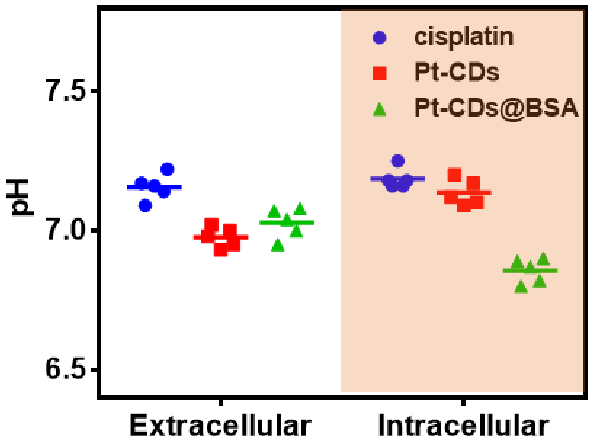

Fig. S21. Effect of cisplatin, Pt-CDs and Pt-CDs@BSA on pHi and pHe. 4T1 cells were loaded with the fluorescent pH indicator BCECF-AM and incubated with cisplatin, Pt-CDs and Pt-CDs@BSA at the Pt concentration of 10 μM with a 589 nm light irradiation (0.5 W/cm^2, 10 min).

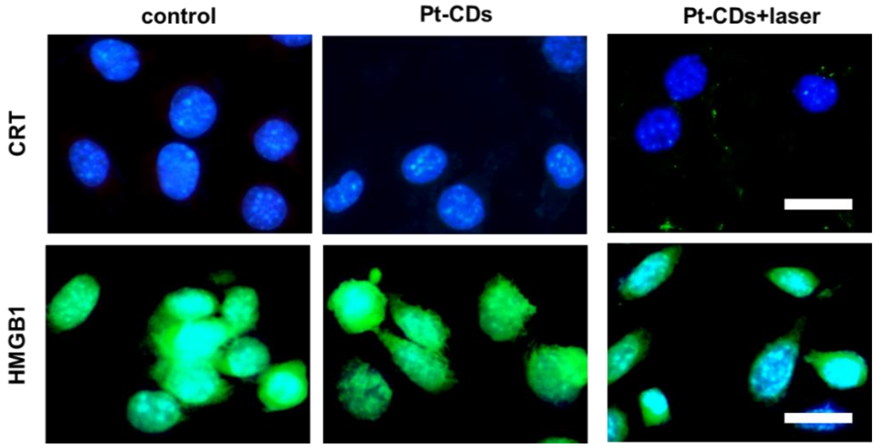

Fig. S22. CLSM images of surface-exposed CRT on 4T1 cells and HMGB1 release from 4T1 cells treated with Pt-CDs@BSA at the Pt concentration of 10 μM with and without 589 nm laser irradiation (0.5 W/cm^2, 10 min). Scale bar= 20 μm.

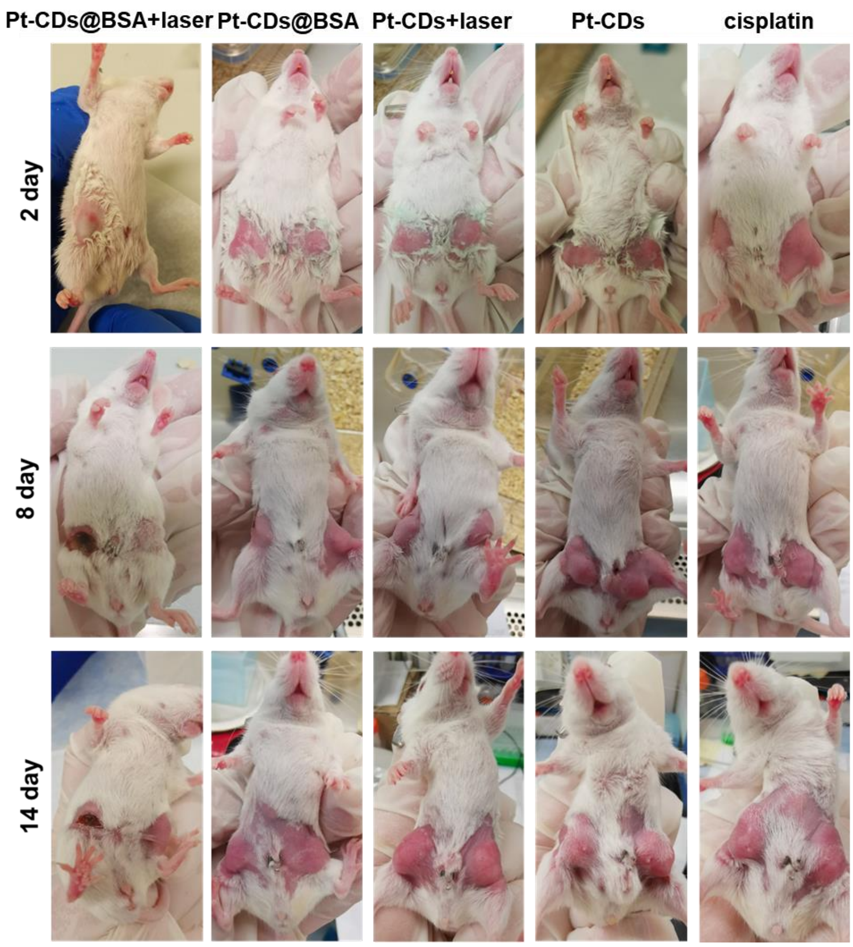

Fig. S23. Typical images of the 4T1 tumor bearing mice treated with Pt-CDs@BSA+laser, Pt-CDs@BSA, Pt-CDs+laser, Pt-CDs, and cisplatin at Day 2, 8, and 14.

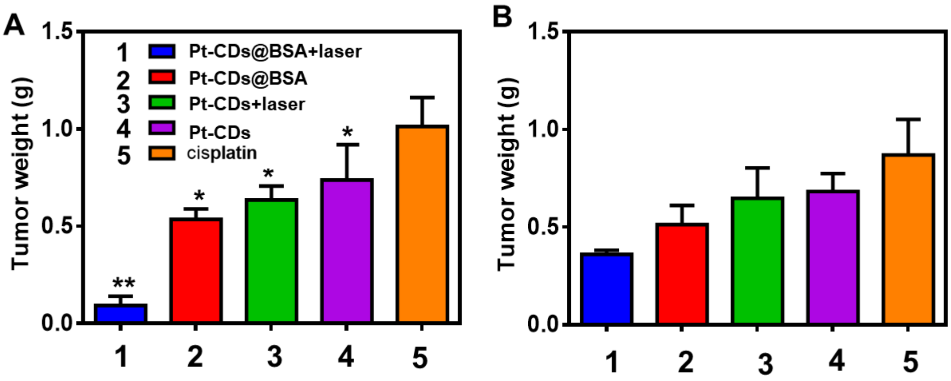

Fig. S24. (A) Primary tumor and (B) distant tumor weight at the end of treatment experiment. Error bars indicate standard deviations (n = 5).

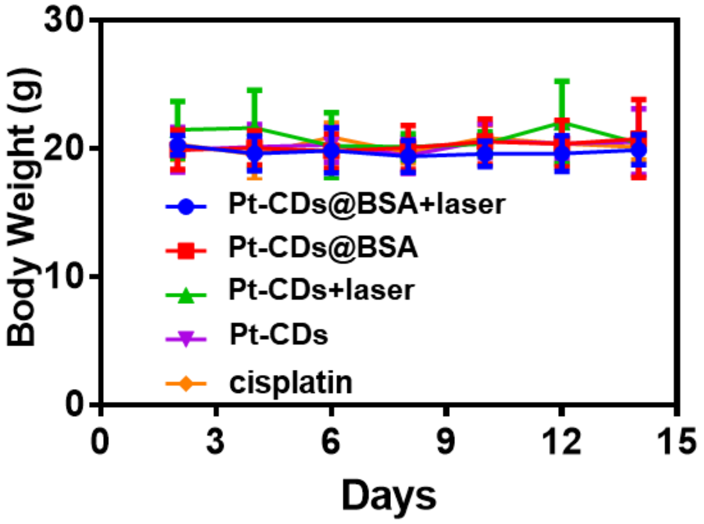

Fig. S25. Body weight variation of tumor-bearing mice during the treatment. Error bars indicate standard deviations (n = 5).

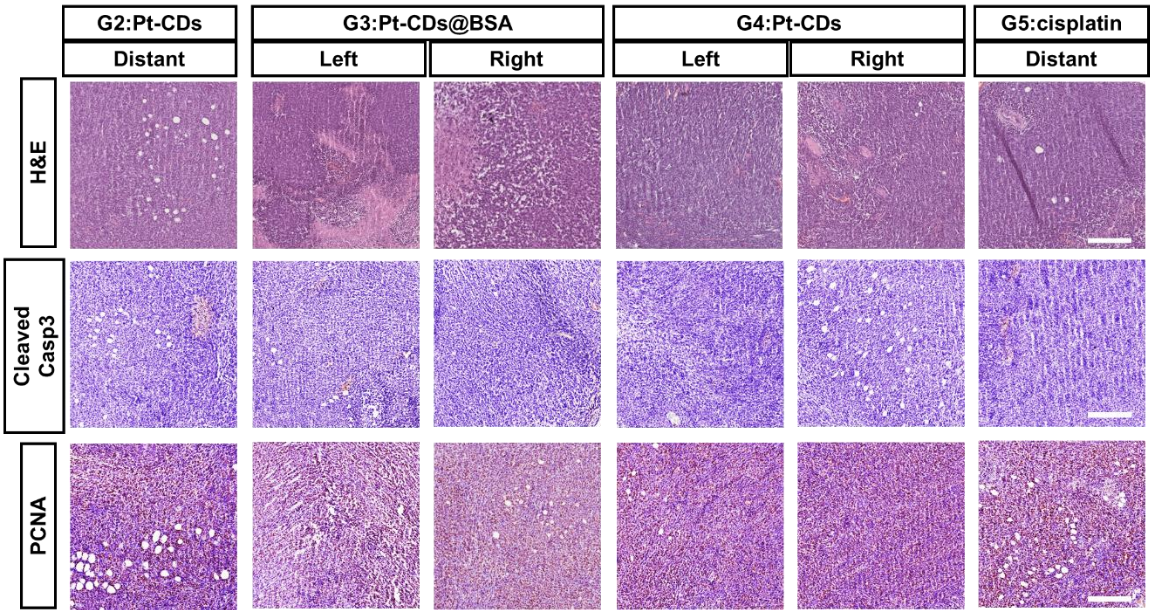

Fig. S26. H&E, cleave caspase 3, and PCNA staining of the tumors after one-round of G2, G3, G4, and G5 group treatment. Scale bar: 250 µm.

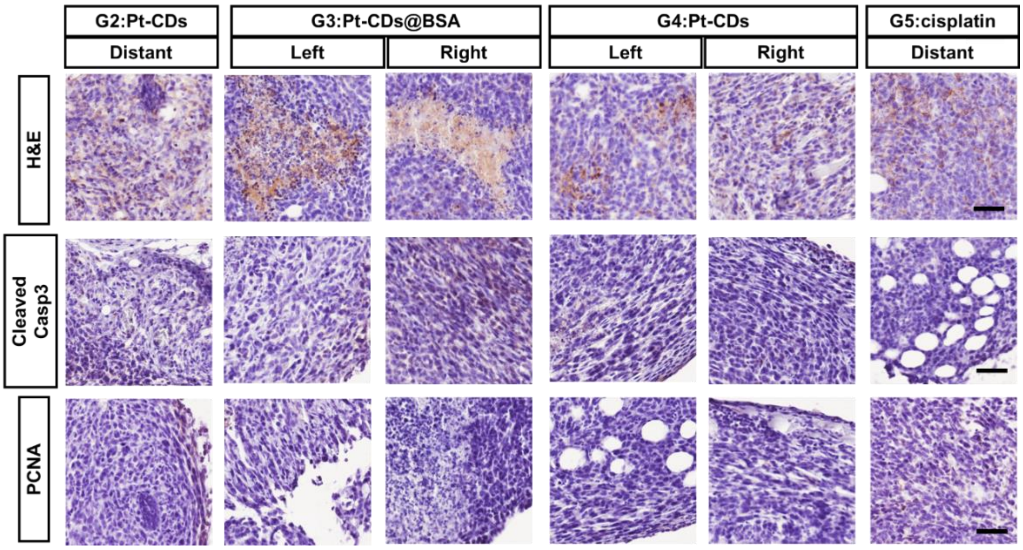

Fig. S27. $CD3^+$, $CD4^+$, and $CD8^+$ immunohistochemical staining of tumor in G2, G3, G4, and G5 group. Scale bar: 250 μm.

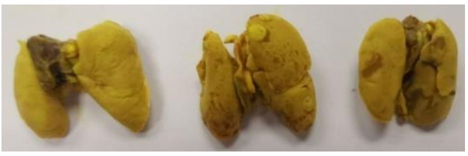

Fig. S28. Representative photographs of lungs with metastatic nodules stained by Bouin's fluid for group of the Pt-CDs@BSA+laser.

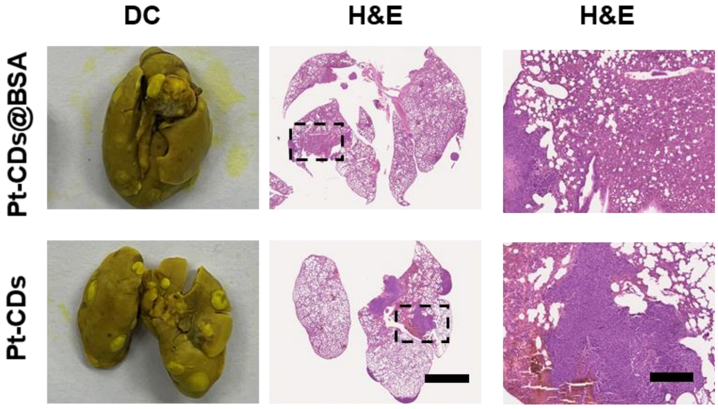

Fig. S29. Digital photographs of lungs with metastatic nodules stained by Bouin's fluid and H&E staining of lung sections from 4T1-tumor-bearing mice treated with Pt-CDs and Pt-CDs@BSA. The red circles indicate metastasis nodules. Scale bar: 2.5 mm in low resolution and 1.0 mm in high resolution.

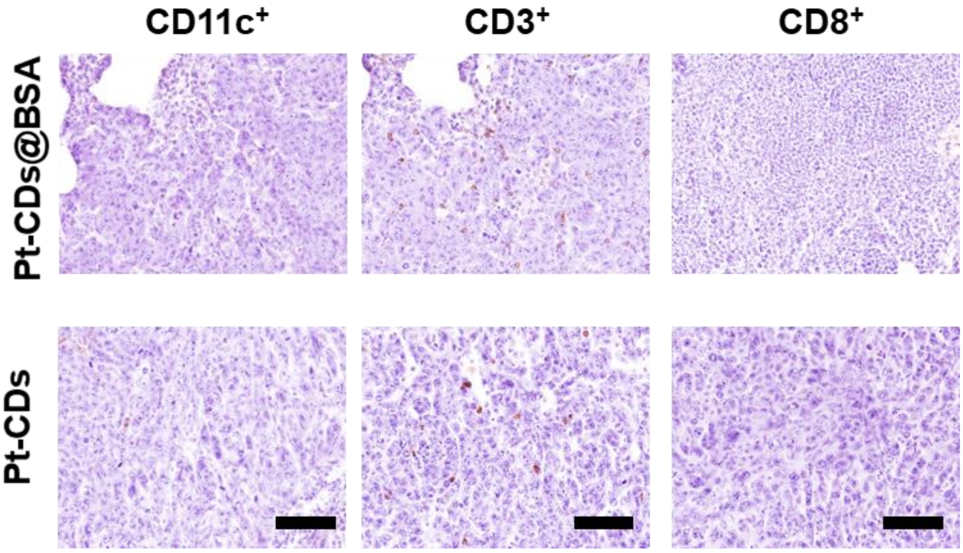

Fig. S30. CD11c$^+$, CD3$^+$, and CD8$^+$ immunohistochemical staining of lungs in each group. Scale bar: 50 μm. Injection dose: 2.5 mg Pt/kg.

Table S8. Antibodies used for experiments.

Item Description	Source	Model / Cat. #	IHC/IF
Cleaved Caspase-3 (Asp175) Antibody	CST	#9661	1:1000
CD8α (D8A8Y) Rabbit mAb	CST	#85336	1:400
CD11c (D1V9Y) Rabbit mAb	CST	#97585	1:400
CD3e Monoclonal Antibody (145-2C11)	CST	#53-0031-82	1:1000
F4/80 Monoclonal Antibody (BM8)	eBioscience™	#14-4801-82	1:100
Anti-PCNA antibody	Abcam	#ab92552	1:100
HMGB1 Polyclonal Antibody	ThermoFisher Scientific	#PA1-16926	1:500
Calreticulin Polyclonal Antibody	ThermoFisher Scientific	#PA5-25922	1:500
Donkey anti-Rabbit IgG (H+L) Highly Cross-Adsorbed Secondary Antibody, Alexa Fluor™ Plus 488	ThermoFisher Scientific	# A32790	1:1000
